# Zinc nanoparticles ameliorated obesity-induced cardiovascular disease: role of metabolic syndrome and iron overload

**DOI:** 10.1038/s41598-023-42550-y

**Published:** 2023-09-25

**Authors:** Samir A. E. Bashandy, Ahmed M. A. El-Seidy, Fatma A. A. Ibrahim, Sahar S. Abdelrahman, Sherif A. Abdelmottaleb Moussa, Marawan A. ElBaset

**Affiliations:** 1 Pharmacology Department, National Research Centre, 33 El-Bohouth St., Dokki, P.O. 12622, Cairo, Egypt; 2 Inorganic Chemistry Department, National Research Centre, 33 El-Bohouth St., Dokki, P.O. 12622, Cairo, Egypt; 3 Biophysics Group, Department of Biochemistry, National Research Centre, 33 El-Bohouth St., Dokki, P.O. 12622, Cairo, Egypt; 4 Pathology Department, Faculty of Veterinary Medicine, Cairo University, Cairo, Egypt

**Keywords:** Biochemistry, Cardiology, Diseases, Health care

## Abstract

Obesity is a complicated disease characterized by abundant fat accumulation. It is associated with cardiovascular disease. The current study aimed to appreciate the role of synthesized zinc oxide nanoparticles (ZnONPs) (18.72 nm in size) in curbing cardiovascular disease in an obesity model of a high fat/sucrose diet in male rats. For 16 weeks, 24 rats were fed a high-fat diet and a 25% sucrose solution to develop obesity, and after that, the rats were randomly allocated into four groups of rats. Group 1 served as the control group and consisted of normal, non-obese rats. Group 2 comprised obese rats that were injected with an equivalent volume of a neutral substance, serving as vehicle control. In Group 3 or 4, obese rats were treated with an intraperitoneal injection of 5 or 10mg/kg of zinc oxide nanoparticles (ZnONPs) for eight weeks. The treatment of obese rats with ZnONPs decreased plasma levels of monocyte chemoattractant Protein-1 (MCP-1), resistin, ENA78, tumor necrosis factor-alpha (TNF-α), interleukin 6 (IL6), and C reactive protein (CRP). Also, the remediation of obese rats with ZnONPs led to a significant decrease in body mass index (BMI), body weight gain, leptin, cholesterol, triglycerides, LDL (Low-density lipoprotein), glucose, and insulin resistance index (HOMA-IR). Moreover, ZnONPs treatment lowered troponin, creatine phosphokinase-MB (CK-MB), lactate dehydrogenase (LDH), cardiac or adipose tissue iron content, and malondialdehyde (MDA) either in blood or heart tissue. Otherwise, treating obese rats with ZnONPs enhanced plasma adiponectin levels, cardiac-reduced glutathione (GSH), and superoxide dismutase (SOD). In addition, ZnONPs displayed a significant influence on the cardiovascular system since they combat the rise in blood pressure and the pathological changes of the heart and aorta besides maintaining plasma nitric oxide levels. The results showed a positive correlation between BMI and MDA, MPC-1, CK-MB, and LDH. ZnONPs are convenient in treating cardiovascular disease in obese rats via reduced blood pressure, oxidative stress, cardiac iron accumulation, insulin resistance, and inflammatory markers.

## Introduction

Obesity is viewed as a risk agent for several metabolic illnesses, such as hypertension, dyslipidemia, and diabetes, which are closely related to the growth of cardiovascular diseases (CVDs) that lead to abnormalities of the heart and blood vessels^1,2^. Obesity is linked with an increased chance of developing insulin resistance that collaborates to generate CVD via atheroma plaque development, ventricular hypertrophy, and diastolic abnormalities^3^. Free radical-induced tissue damage may affect obesity and related diseases^4^. Reactive oxygen species (ROS) evoke inflammation and lipid peroxidation that may consider risk factors in CVD^5^. Increased inflammatory processes and ROS may be implicated in dietary saturated fatty acid-caused CVDs^6^. A fat-rich diet is a potent ROS stimulator that modifies oxidation metabolism. The fat accumulation from a high-fat diet is liable to lipid peroxidation in developing atherosclerosis^7^. According to research, “lipid oxidation, protein glycation, glucose auto-oxidation, and the production of lipid peroxidation” metabolites promote tissue damage from obesity-related dyslipidemia^8^. Increased ROS markers and byproducts in cardiac tissue injure cells directly^9^. Extreme accumulation in adipose tissue of obese patient result in elevation of blood pressure as a consequence of stimulation of the renin–angiotensin–aldosterone system^10^. Decreased nitric oxide bioavailability and increased intima-media thickness may result in endothelial dysfunction in obesity^11–13^. Adiponectin lowers vascular and intracellular adhesion molecule expression, suggesting adipocyte hormones fight cardiovascular disease. Leptin and MCP-1 (Hyperleptinemia) have been associated with developing hypertension and endothelial dysfunction^14^. Increased adiposity is associated with dysregulations in iron homeostasis, presenting as increased serum hepcidin, elevated serum ferritin, and an increased risk of iron overload, with potential implications in impairments in metabolic health^15^.

Nanotechnology has advanced rapidly globally, and manufactured nanoparticles are used in paints, cosmetics, medicines, food, electronics, and clothes due to their small size and unique features^16–19^. ZnONPs are significant nanoparticles because of their physical and chemical characteristics. Furthermore, ZnONPs have become popular in biological applications due to their low toxicity^20^. ZnONPs have a positive role in biomedicine, particularly in the antifungal, antibacterial, anticancer, and antidiabetic fields^21–23^. Moreover, ZnONPs showed hypolipidemic, antioxidant, and anti-inflammatory properties^24,25^. Zinc is needed for immunity, tissue development, brain function, pathogen defense, and membrane structure and function^26,27^. Moreover, ZnONPs are used in current drug dg-loading capacity, programmable drug release ability, and targeted delivery^28^. Also, zinc has fundamental roles in forming and functioning some antioxidant enzymes and different metallothioneins^29^. Generally, fluctuation in Zn homeostasis participates notably in the progress of CVDs, such as myocardial infarction, coronary heart disease, and ischemic cardiomyopathy, that may lead to CVD mortality^30^. A literature survey suggests zinc supplementation's potential and promising role in obesity-related cardiovascular disease. Based on our knowledge, up to now, there has not been a report on the effect of zinc nanoparticles on obesity-induced cardiovascular disease concerning iron overload, blood pressure, insulin resistance, adipocyte hormones, and inflammatory markers. Consequently, the current investigation was carried out to understand the therapeutic potential of ZnONPs in reversing obesity-related cardiovascular abnormalities in rats by investigating the previous parameters and joining them with oxidative stress.

## Material and methods

### Synthesis of ZnO

The ZnONPs were produced by the sonication-^31^. 5 g (22.78 mmol) Zinc acetate dihydrate (Zn(CH_3_COO)_2_.2H_2_O) was mixed mechanically with 0.45 g (4.03 mmol)) potassium hydroxide pellets (KOH), then added to PEG solution (250 mL DD, 0.23 g of PEG) under sonication (direct immersion, Ultrasonics Vibra cell, 20 kHz, 50% of 550 wt., temperature below 80 °C) for 30 min. The mixture was put under stirring (1000 rpm) at 80 °C. The resulting solution was evaporated entirely. The resulting powder was washed three times with DDW, then dried before calcined at 600 °C for four hours in an air environment in a furnace, and finally crushed.

### Characterization and testing

The work was performed as per Elseidy et al., in which “the XRD patterns were obtained from an X'pert PRO diffractometer with a Cu-radiation (λ = 1.542 Å) at 45 K.V. and 35 mA over the range of 2θ = 5°–80°, and the average size of the crystallites was calculated by Debye–Scherrer equation. The powder's morphology, surface structure, and size were examined under a transmission electron microscope. HR-TEM was carried out using the TEM model JEOL 2100 LB6 transmission electron microscope at the National Research Center, Cairo, Egypt. XPS was collected on K-ALPHA (Thermo Fisher Scientific, USA) with monochromatic X-ray Al K-alpha radiation -10 to 1350 e.v spot size 400 micro m at pressure 10–9 mbar with full spectrum pass energy 200 e.v and at narrow spectrum 50 e.v^31^.

### Experimental protocol

Thirty-two male Wistar rats (age of 10 weeks, weighing 138–155 g) were brought from the Animal house of the National Research Center, Egypt. The rats were indwelled in polyethylene veterinary cages at a stationary room temperature 25C^o^ with 12 h light/dark cycles. Twenty-four rats were given a high-fat diet and 25% sucrose solution for 16 weeks to develop obesity. The high-fat diet consists of carbohydrates 42.3%, protein 17%, fat 22.50%, fiber 3.2%, minerals 5%, and moisture 10% (Moreno-Fernández et al.,2018). Normal rats (8) were fed free standard chow pellets: “fat 5%, carbohydrates 65%, proteins, 20.3% fiber 5%, salt mixture 1%and vitamin mixture 3.7%”^1^.

**Figure 1:**
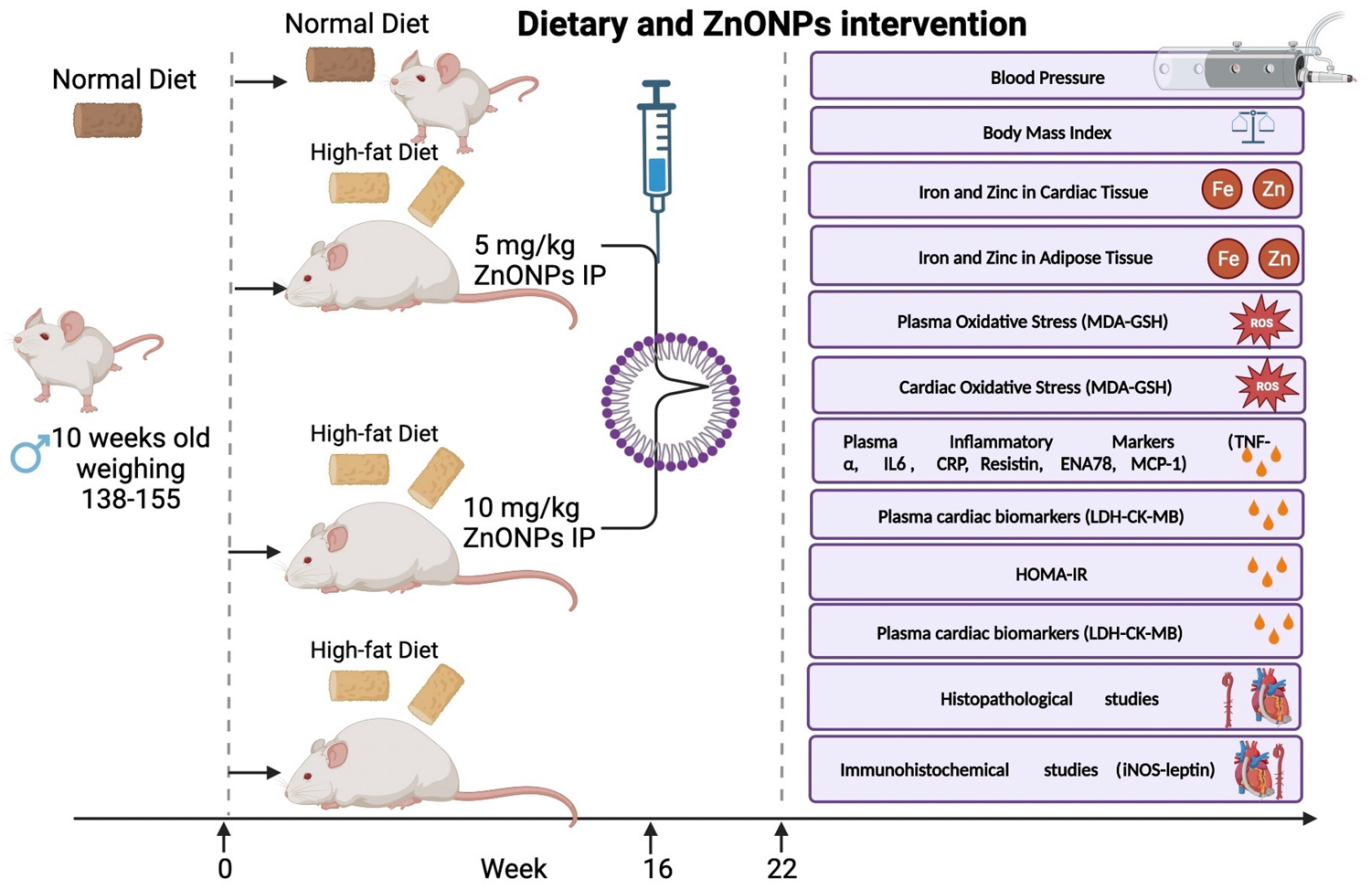
Experimental design of the Zinc nanoparticles intervention in obese rats.

Rats that weren't overweight served as the "control" in Group 1. After 16 weeks of being given a high-fat diet and a 25% sucrose solution to induce obesity, 24 rats were randomly split into three groups. The second group consisted of obese rats that had a same amount of a neutral chemical injected into them as a control obese. For eight weeks, rats in Groups 3 and 4 were given injections of either 5 or 10mg/kg of zinc oxide nanoparticles (ZnONPs) intraperitoneally as shown in Fig. 1. The doses of ZnO NPs used were according to previous work^32^.

Food consumption was calculated daily by subtracting the amount put over each cage from the measured amount of food that remained the next day (gm/day/rat). The mean food consumption per rat was calculated. ZnO NPs were suspended in distilled water.

### Anthropometric measures

Body mass index (BMI) can be calculated concerning the height and weight of a rat according to the formula: “BMI = body weight (g)/height (cm^2^)”. The circumference of the waist was measured. BMI was recorded.

### Samples

At the end of the treatment period, the rats of all groups were fasted for about 12 h. “Blood samples were collected from a tail vein in heparinized tubes, and separated plasma was stored in Eppendorf tubes at -80˚ for biochemical analysis”^33^ under light anesthesia with ketamine.

Animals were killed by cervical dislocation while sedated with ketamine, and their hearts and aortas were removed just after blood was drawn. “A weighted part of the heart was homogenized with ice-cooled saline (0.9% NaCl) to prepare homogenate. The homogenate was then centrifuged at 3000 rpm for 10 min. At 5 °C using a cooling centrifuge “Laborzentrifugen, Sigma, Germany”. The supernatant was used for various analyses. A portion of the heart and aorta were fixed immediately in 10% neutral buffered formalin, processed for light microscopy to get (5 μm) paraffin sections, and stained with “Hematoxylin & Eosin (H &E)” to verify histological details. Furthermore, immunochemistry was performed on certain heart sections, company, Germany)”^33^.

### Determination of iron and zinc in tissues

Iron was determined in both adipose and cardiac tissues according to the method described by Imeryuz et al. (2007). “Briefly, tissue(1 g) was treated with 65% nitric acid for 30 min and then with 60% perchloric acid. After centrifugation (3000 g for 12 min), the supernatant was diluted with deionized water, and non-heme iron was determined by graphite furnace atomic absorption spectrophotometer and expressed as mg non-heme iron/g of tissue weight”^34,35^.

### Plasma and cardiac oxidative stress parameters

In this study, we employed well-established colorimetric assays to determine the levels of Malondialdehyde (MDA), Superoxide Dismutase (SOD), and Glutathione (GSH) in both plasma samples and heart tissues.

#### Malondialdehyde (MDA) assessment

Malondialdehyde is a widely used marker of lipid peroxidation, reflecting the extent of oxidative stress in biological systems. We followed the thiobarbituric acid reactive substances (TBARS) assay to quantify MDA levels in plasma. Briefly, plasma samples were mixed with thiobarbituric acid (TBA) solution, and the resulting MDA-TBA adduct was measured spectrophotometrically at a specific wavelength. The absorbance was compared against standard MDA solutions, and the MDA concentration was calculated.

#### Superoxide dismutase (SOD) activity measurement

Superoxide Dismutase is a crucial antioxidant enzyme that is key in neutralizing superoxide radicals. To assess SOD activity in plasma and heart tissues, we utilized the Nitroblue Tetrazolium (NBT) reduction assay. This assay is based on the ability of SOD to inhibit the reduction of NBT by superoxide radicals. The rate of NBT reduction was measured at a specific wavelength, and SOD activity was determined by comparing the results with a standard curve generated from known SOD concentrations.

#### Glutathione (GSH) quantification

Glutathione is a vital endogenous antioxidant that protects cells from oxidative damage. In this study, we measured GSH levels in both plasma and heart tissues using a DTNB-GSSG reductase recycling method. Briefly, GSH reacts with 5,5'-Dithiobis(2-nitrobenzoic acid) (DTNB) to produce a colored compound, and the increase in absorbance was monitored spectrophotometrically. The GSH concentration was calculated based on a standard curve generated from known GSH concentrations.

It is important to note that all assays were performed using Biodiagnostic kits from Egypt, ensuring standardized and reliable procedures for the assessment of oxidative stress markers and antioxidants in our samples. The utilization of these established colorimetric assays allows for accurate and reproducible measurements of MDA, SOD, and GSH levels, providing essential insights into the oxidative status of the plasma and heart tissues under investigation.

### Plasma inflammatory markers

In our study, the levels of various inflammatory markers were assessed using the enzyme-linked immunosorbent assay (ELISA) technique, employing the Sunlong Biotech Co. Kit from China. The following inflammatory markers were measured:

- Tumor Necrosis Factor-alpha (TNF-α): TNF-α is a pro-inflammatory cytokine known for its role in promoting inflammation and regulating immune responses. Its quantification allows us to evaluate the extent of inflammatory activity in the samples.
- Interleukin-6 (IL6): IL6 is another key pro-inflammatory cytokine involved in various inflammatory processes. Measuring IL6 levels helps us understand the extent of immune system activation and inflammation.
- C-reactive protein (CRP): CRP is an acute-phase protein produced by the liver in response to inflammation. It serves as a sensitive marker of systemic inflammation and can provide valuable information about the presence and severity of inflammatory conditions.
- Resistin: Resistin is an adipokine that has been associated with insulin resistance and inflammation. Its measurement aids in assessing the role of adipose tissue in inflammatory responses and metabolic disorders.
- Epithelial Neutrophil-Activating Protein 78 (ENA78): ENA78 is a chemokine involved in recruiting neutrophils to sites of inflammation. Evaluating ENA78 levels helps us understand the chemotactic response and the recruitment of immune cells during inflammation.
- Monocyte Chemoattractant Protein-1 (MCP-1): MCP-1 is a chemokine responsible for attracting monocytes to inflamed tissues. Its quantification provides insights into the recruitment of monocytes and their role in inflammation.

The ELISA sandwich technique is a highly sensitive and specific method widely used for the quantification of various biomolecules, including cytokines and other inflammatory markers. It relies on the specific binding of antibodies to the target molecules, allowing for accurate and precise measurements of their concentrations.

By utilizing the Sunlong Biotech Co. Kit from China, we ensured standardized and reliable measurements of these inflammatory markers. The ELISA technique's robustness and reproducibility will contribute to the validity of our findings and support the significance of these inflammatory markers in our study.

### Plasma biomarkers for myocardial function

In our study, we employed specific immunoassay techniques to evaluate Troponin levels. The Troponin analysis was conducted using a highly reliable and validated kit provided by Sunlong Biotech Co. (China). This kit is well-known for its accuracy and sensitivity in detecting troponin, which is an essential biomarker for assessing cardiac health.

Furthermore, we assessed Creatinine Kinase-MB (CK-MB) and lactate dehydrogenase (LDH) activities using a kinetic method. For the CK-MB analysis, we employed a kinetic assay provided by Centronic-gmbh company (Germany). This particular method allows continuous monitoring of the reaction, enabling us to obtain precise and time-dependent measurements of CK-MB activity. Centronic-gmbh is renowned for producing high-quality diagnostic tools, and their kinetic assay has been widely used in research and clinical settings.

Likewise, for the measurement of lactate dehydrogenase activity, we utilized another kinetic assay from Centronic-gmbh. This approach enabled us to accurately assess the activity of LDH in our samples over a specific time period, providing valuable insights into potential cellular damage or other physiological conditions.

### The adipocyte hormones and lipid profile

In our study, we quantified the plasma levels of leptin and adiponectin using a highly sensitive and specific immunoassay method known as ELISA. For the analysis, we employed ELISA kits provided by Sunlong Biotech Co., China, with the following catalogue numbers: E-CL-R0416 for leptin and E-OSEL-R0006 for adiponectin. ELISA is a widely recognized and widely used technique for detecting and measuring proteins, and it allowed us to accurately assess the concentrations of leptin and adiponectin in the plasma samples.

To determine the lipid profile, specifically cholesterol, triglycerides, HDL (High-Density Lipoprotein), and LDL (Low-Density Lipoprotein), we utilized colorimetric evaluation. For this purpose, we utilized kits manufactured by the reputable Salucea Company, Netherlands. These kits are well-established in lipid analysis and offer high reliability and precision in lipid parameter measurements. The colorimetric method is based on the formation of color complexes proportional to the concentration of the respective lipid, which we quantified using a spectrophotometer.

Furthermore, to assess the atherogenic index, a crucial indicator of cardiovascular risk, we employed a straightforward and commonly used formula: Atherogenic Index (AI) = log (Triglycerides/HDL-Cholesterol). This formula allowed us to calculate the atherogenic index and obtain insights into the potential risk of atherosclerosis and cardiovascular complications.

### Insulin resistance parameters

#### Colorimetric analysis of blood glucose

Blood glucose levels were determined through a colorimetric analysis using a highly reliable and validated kit from the reputable Salucea Company. The colorimetric analysis involves the use of specialized reagents and equipment to measure the concentration of glucose in the plasma accurately. This method is based on the enzymatic reaction between glucose and specific reagents, leading to the formation of a colored product whose intensity is directly proportional to the glucose concentration. The absorbance of the colored product was measured at a specific wavelength using a spectrophotometer, allowing us to quantify the blood glucose levels with precision.

### Measurement of insulin levels in plasma

Insulin levels in the plasma were quantified using a robust enzyme-linked immunosorbent assay (ELISA) kit sourced from Sunlong Biotech Co. in China. The ELISA technique is widely recognized for its sensitivity and specificity in detecting and measuring the concentration of target molecules, such as insulin, in biological samples. This assay employs specific antibodies that bind selectively to the insulin molecules present in the plasma. After a series of carefully controlled washing steps, the bound insulin is detected by adding a substrate that produces a detectable signal, typically a colored product, in proportion to the amount of insulin present. The absorbance of the colored product is measured using a microplate reader, enabling us to accurately quantify the insulin levels in the plasma.

### Calculation of insulin resistance index (HOMA-IR)

The insulin resistance index (HOMA-IR) was calculated using the following widely accepted equation:

$${\text{Insulin}}\;{\text{ resistance}}\;{\text{ (HOMA-IR)}} = \left( {{\text{Fasting}}\;{\text{ glucose}}\;{\text{ in }}\;{\text{mg/dl}}} \right) \times \left( {{\text{Fasting}}\;{\text{ insulin}}\;{\text{ in }}\;{\text{mIU/ml}}} \right)/405$$

HOMA-IR is a valuable indicator of insulin resistance, providing insights into the body's ability to respond to insulin. It is derived from fasting glucose and fasting insulin levels, both of which were obtained through the aforementioned methods.

### Measurement of blood pressure

Blood pressure measurements were taken using the tail-cuff method^36^ with the CODA System (Hakubatec Life Science Solutions, Tokyo, Japan). The tail-cuff method is a widely accepted non-invasive technique for assessing blood pressure in small laboratory animals.

To measure the systolic and diastolic blood pressure, each animal was gently restrained, and the tail-cuff was placed around the tail. The tail-cuff was inflated to temporarily occlude blood flow, and the CODA System recorded the pressure changes as the cuff gradually deflated. This process allowed us to obtain readings for both systolic and diastolic blood pressure.

To ensure accuracy and reliability, blood pressure measurements were taken three times for each animal, and the average value of these three readings was calculated. All measurements were performed at a fixed time window, specifically between 11:00 am and 03:00 pm, to minimize potential variations due to circadian rhythms.

The use of the CODA System, known for its precision and consistency, coupled with the standardized measurement protocol, allowed us to obtain reliable blood pressure data for our study subjects.

### Histopathological studies

The excised cardiac and abdominal aortae tissue specimens were wiped of blood and immediately fixed in buffered neutral formalin. After 24 h, the specimens were routinely processed for getting paraffin blocks, which then were serially sectioned into 4–5 μm thick sections. The later sections were stained with hematoxylin, eosin (H&E), Orcein, and Gomori’s Trichrome, according to^37^.

### Immunohistochemical studies

The Avidin–biotin peroxidase technique (DAB, Sigma Chemical Co.) was employed to detect iNOS expression in cardiac tissues across all groups using immunohistochemical methods. Paraffin sections were treated with a monoclonal iNOS antibody (Dako Corp, Carpenteria, CA) at a 1:200 dilution, along with the recommended reagents for the avidin–biotin-peroxidase technique (Vactastain ABC peroxidase kit, Vector Laboratories). The marker expression was visualized using Chromagen 3,3-diaminobenzidine tetrahydrochloride (DAB, Sigma Chemical Co.). To quantitatively analyze the optical density of the positive brown color in 5 microscopic fields, image analysis software (Image J, 1.46a, NIH, USA) was utilized.

The scoring of periaortic fat leptin expression in different types of adipocytes (unilocular white adipocytes, multilocular brown adipocytes, and differentiating adipocytes) was categorized into four levels of staining intensity as follows: leptin negative (0), weakly leptin positive (I), leptin positive (II), and intensely leptin positive (III).

### Aortic Morphometric study

The thickness of tunica intima and media, as well as the adventitia, was measured using Image J software analysis (Image J, 1.46a, NIH, USA).

### Ethical approval

Animal handling was carried out according to recommendations and under the regulations of Animal Care and Use of the National Research Centre in Egypt with ethical approval No.19218. All surgery was performed under anesthesia, and all efforts were made to reduce suffering. Also, the studies were also carried out in compliance with the ARRIVE guidelines and the animal welfare compliance Guide for the Care and Use of Laboratory Animals (8thed., 2011).

## Results

### ZnO nanocomposite characterizations

**Figure 2:**
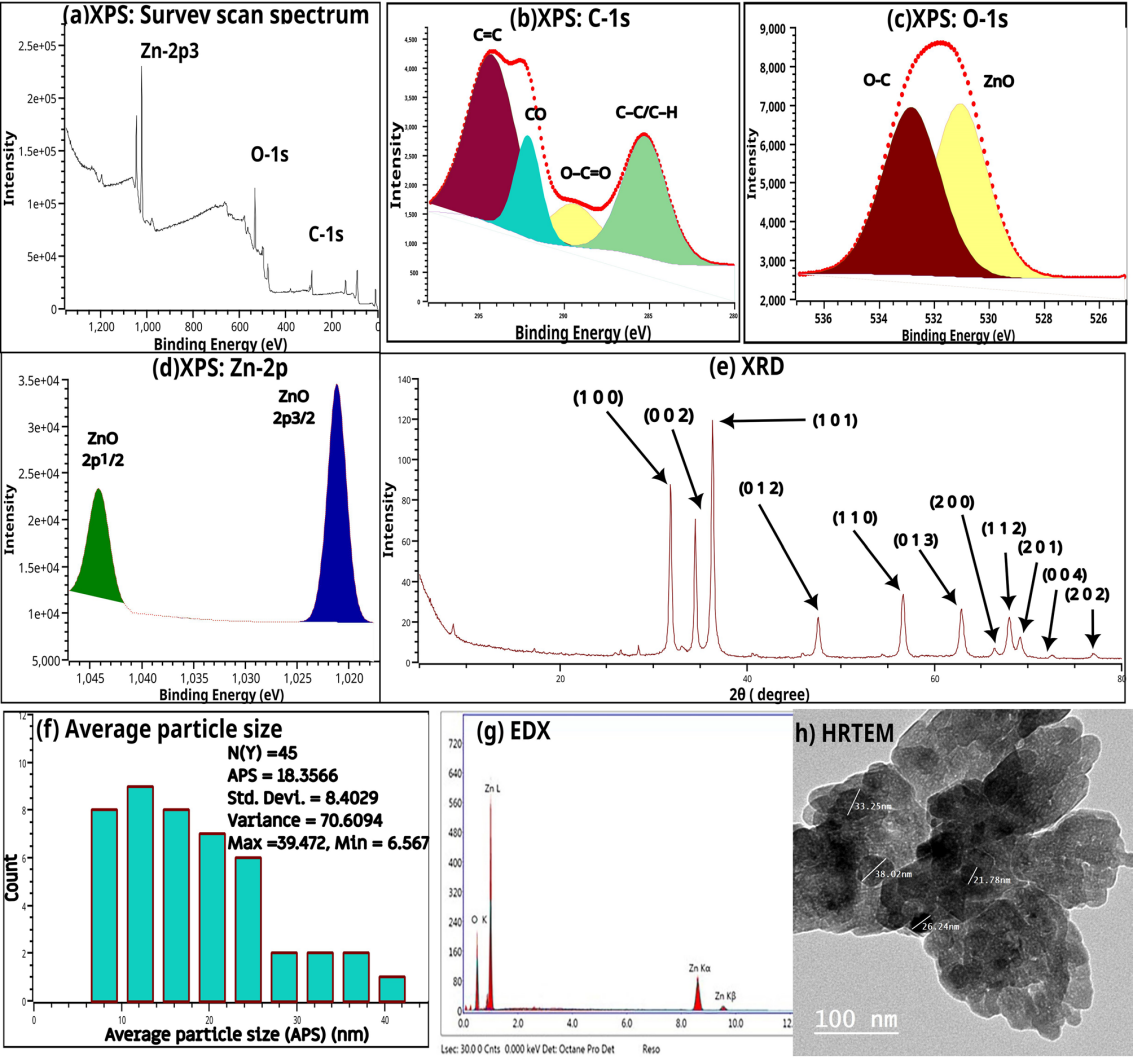
(**a**) XPS Survey scan spectra of nano zinc oxide, (**b**) C-1s XPS spectra, (**c**) O-1s XPS spectra, (**d**) Zn-2p XPS spectra, (**e**) XRD patterns of nano zinc oxide, (**f**) Particle diameters distributions, (**g**) EdX and (**h**) HRTEM of nano zinc oxide.

Figure 2a illustrates the survey scan spectrum of nano zinc oxide. The survey indicated the presence of C-1s, Zn-2p, and O-1s. Figures 2b–d showed the deconvoluted spectrum of C-1s, O-1s, and Zn-2p, respectively. The XPS spectrum of C-1s showed peaks at 285.27, 289.48, 292.15, and 294.28, while that of ZnO deconvoluted into ZnO 2p_5/2_ (1044.19 eV) and ZnO 2p_3/2_ (1021.24). The XPS spectrum of O-1s showed two peaks at 531.06 eV and 532.84 eV. Figure 2e illustrates the XRD patterns of the nano zinc oxide. This figure show diffraction peaks at 2θ of 31.74°, 34.39°, 36.23°, 47.50°, 56.55°, 62.80°, 66.32°, 67.89°, 69.03°, 72.49° and 76.89° which are indexed to (1 0 0), (0 0 2), (1 0 1), (0 1 2), (1 1 0), (0 1 3), (2 0 0), (1 1 2), (2 0 1), (0 0 4) and (2 0 2), respectively. The calculated parameters include cell parameters (a = b = 32.52 × 10^–2^ nm, c = 52.11 × 10^–2^ nm and c/a = 1.60), axial ratio (a.r.) = 160.24 × 10^–2^, unit cell volume (V) = 4.77 × 10^–2^ nm^3^, oxygen-position parameter (u) = 37.98 × 10^–2^, zinc–oxygen bond length (L) = 19.79 X 10^-2^ nm and interplanar spacing (d_100_ = 28.16 × 10^–2^ nm, d_001_ = 26.06 × 10^–2^ nm and d_101_ = 24.78 × 10^–2^ nm). Figure 2f showed the histograms of particles diameters distributions. The average particle diameters are about 18.36 nm (length), while the calculated value of average particle diameters was 18.72. The results of EDX (Fig. 2g) indicates the presence of Zn and oxygen only.

### Anthropometric measures, adipose tissue weight, and food consumption

The treatment of obese rats with ZnONPs (5 or 10 mg/kg) led to a significant decrease (at *p* = 0.0037 or *p* < 0.0001) in body weight compared to the obese group, respectively (Table 1). The percentage of increase in body weight gain for control, obese, and obese treated with 5 or 10 mg/kg ZnONPs were 24%, 43%, 34%, and 19%, respectively. The values of abdominal circumference and BMI of obese rats administered ZnONPs (5 or 10 mg/kg) were significantly lower (at *p* = 0.0001 or *p* < 0.0001) than those of obese rats, respectively. The percentage of change of BMI relative to starting time was 7.27%, 25.00%, 18.00%, and 5.41% for control, obese, O + ZnONPs 5 or 10 mg/kg, respectively. The treatment of obese rats with ZnONPs (5 or 10 mg/kg) gave rise to a significant lowering in food consumption (at *p* = 0.0287 or *p* = 0.0039) and weight of epididymal (at *p* = 0.0022 or *p* = 0.0109) and visceral fats (at *p* < 0.0001 or *p* < 0.0001) (Table1), respectively.

**Table 1:** Anthropometric measures and adipose tissue weight of obese rats treated with zinc oxide nanoparticles.

Parameter		Treatment			
		Control	Obese	O + ZnONPs 5 mg/kg	O + ZnONPs 10 mg/kg
Body weight (g)	Starting	242.00 ± 7.33	341.80 ± 8.65*	320.66 ± 11.34*	331.90 ± 12.69*
	Final	300.78 ± 5.43	489.43 ± 13.15*@	428.36 ± 12. 00 *@	395.74 ± 13.00*@#
	% of change	24.30%	43.19%	33.59%	19.28%
BMI (g/cm^2^)	Starting	0.55 ± 0.011	0.76 ± 0.020*	0.72 ± 0.028*	0.74 ± 0.032*
	Final	0.59 ± 0.010	0.95 ± 0.018*	0.85 ± 0.013*@	0.78 ± 0.012*@#
	% of change	7.27%	25.00%	18.00%	5.41%
Abdominal circumference(cm)	Starting	13.53 ± 0.30	18.63 ± 0.18*	17.63 ± 0.38*	17.81 ± 0.32*
	Final	15.63 ± 0.42	23.13 ± 0.23*	18.63 ± 0.26*@	19.00 ± 0.38*@
	% of change	15.52%	24.15%	5.67%	6.68%
Adipose tissue weight (g)	Epididymal	2.34 ± .0.28	6.40 ± 0.56*	4.35 ± 0.31*@	4.67 ± 0.19*@
	Visceral	2.81 ± 0.26	16.01 ± 0.86*	10.17 ± 0.28*@	8.10 ± 0.29*@#
Food intake (g/day)		13.55 ± 1.00	19.43 ± 0.78*	16.32 ± 0.57*@	15.47 ± 1.00 *@

Each value represents the mean of 8 animals ± SE. Statistical analysis was performed using one-way ANOVA followed by Tukey–Kramer multiple comparisons test (* vs control group, ^@^vs obese group and ^#^vs ZnONPs 5 mg/kg) at *p* < 0.05. O: Obese.

### Lipid profile, adipocyte hormones, and blood pressure

High blood lipid levels and pressure are risk factors for cardiovascular disease. As detected in Table 2, the treatment of obese rats with ZnONPs (5 or 10 mg/kg) significantly decreased cholesterol (at *p* < 0.0001) by 33% or 56%, triglycerides (at *p* < 0.0001 or *p* < 0.0001) by 13% or 27%, LDL (at *p* < 0.0001 or *p* < 0.0001) by 50% or 71%, and the atherogenic index (at *p* < 0.0001 or *p* < 0.0001) by 34% or 50%. At the same time, the HDL level was enhanced (at *p* < 0.0001 or *p* < 0.0001) by 4 or 5.7-fold, compared to the obese group, respectively. Feeding rats with a diet supplemented with high fat and sucrose resulted in a significant increase in leptin levels (at *p* < 0.0001) by 60% and a substantial decrease in adiponectin levels (at *p* < 0.0001) by 73%, compared to the normal group, respectively. In addition, the treatment of obese rats with ZnONPs (5 or 10 mg/kg) decreased the leptin hormones level (at *p* < 0.0001 or *p* < 0.0001) by 30% or 40%, while the adiponectin was raised (at *p* < 0.0001 or *p* < 0.0001) by 3 or 3.9-fold, compared to the obese group, respectively (Table 2). Besides, the systolic and diastolic blood pressure of obese rats was elevated (*p* < 0.0001) by 5 and 1.2-fold. In contrast, the rats with ZnONPs (5 or 10 mg/kg) significantly hampered the systolic (at *p* = 0.0001 or *p* < 0.0001) by 34% or 50% and diastolic blood pressure (at *p* = 0.0002 or *p* < 0.0001) by 14% and 21% (Table 2), respectively.

**Table 2:** Plasma lipid profile (mg/dl), adipocyte hormones (µg/L), nitric oxide (µmol/ml) levels and blood pressure (mmHg) of obese rats treated with zinc oxide nanoparticles.

Treatment Parameter	Control	Obese	O + ZnONPs 5 mg/kg	O + ZnONPs 10 mg/kg
Cholesterol	95.16 ± 2.95	211.5 ± 4.724*	134.1 ± 2.962*^@^	89.75 ± 7.034^@#^
Triglycerides	70.22 ± 1.78	244 ± 14.58*	204.5 ± 15.47*	173.7 ± 13.75*^@^
HDL	39.07 ± 2.44	7.55 ± 0.4734*	29.22 ± 2.45*^@^	41.6 ± 2.576^@#^
LDL	22.76 ± 1.36	92.51 ± 1.68*	44.94 ± 2.362*^@^	26.26 ± 0.9407^@#^
Atherogenic index	0.26 ± 0.03	1.51 ± 0.0416*	0.8492 ± 0.0583*^@^	0.6175 ± 0.0394*^@#^
Leptin	1.87 ± 0.15	3.11 ± 0.15*	2.19 ± 0.25*^@^	1.94 ± 0.19^@#^
Adiponectin	196 ± 10.31	51.13 ± 1.49*	151.88 ± 8.33*^@^	188.13 ± 8.80^@#^
Nitric oxide	40.80 ± 1.16	17.88 ± 0.78*	26.00 ± 1.05*	38.50 ± 1.68^@#^
Systolic BP	117.87 ± 2.13	145.76 ± 2.95*	130.38 ± 1.06*^@^	121.62 ± 1.89 ^@^
Diastolic BP	81.13 ± 1.62	102.50 ± 2.60*	88.38 ± 1.44^@^	80.75 ± 2.23^@^

Each value represents the mean of 8 animals ± SE. Statistical analysis was performed using one-way ANOVA followed by Tukey–Kramer multiple comparisons test (*vs control group, ^@^vs obese group and ^#^vs ZnONPs 5 mg/kg) at *p* < 0.05. O: Obese.

### Inflammatory markers

**Figure 3:**
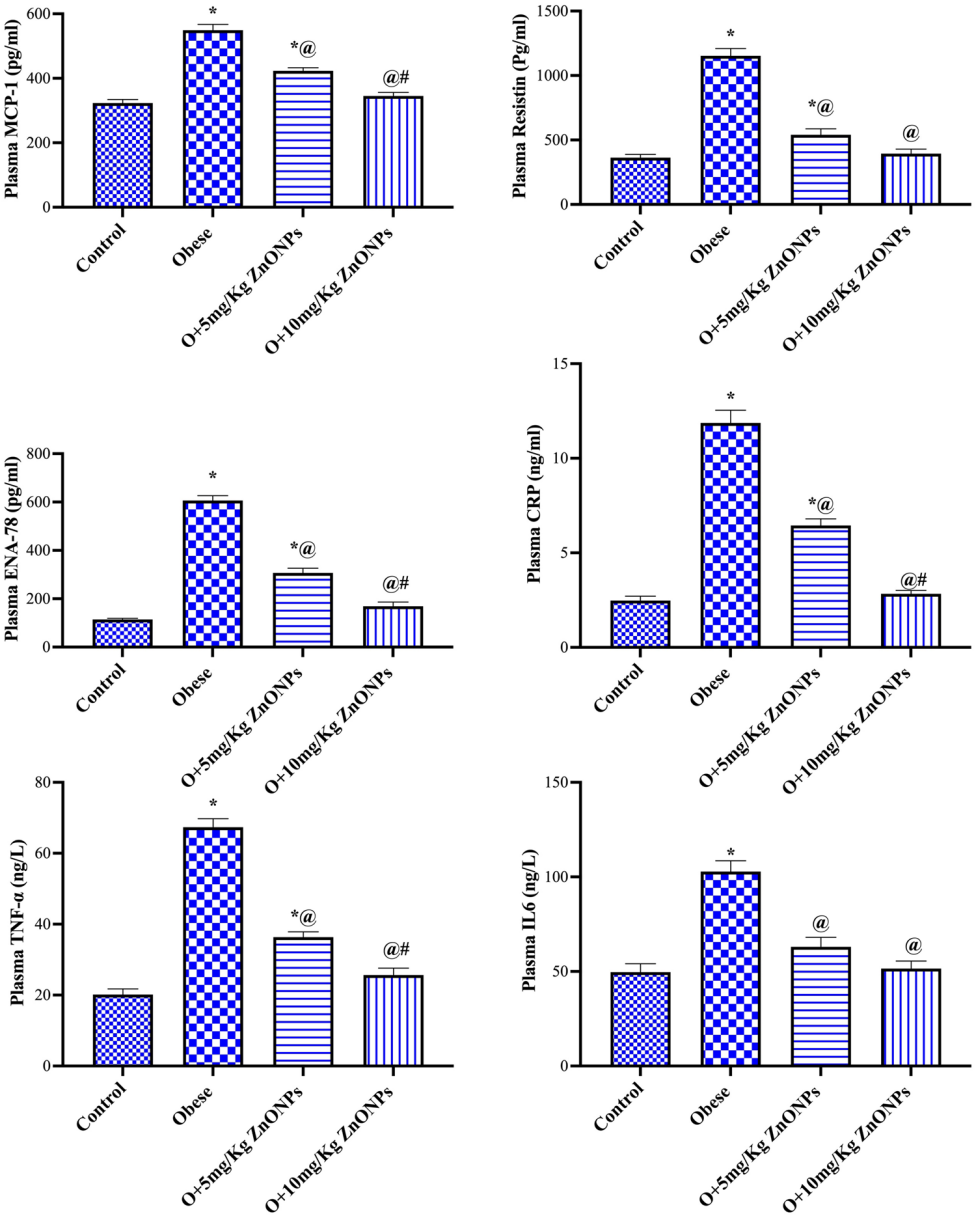
Effect of ZnONPs on plasma level of MPC-1, resistin, ENA-78, TNF-α, IL6, and CRP in obese rats. Each bar represents the mean ± SE of 8 rats. *vs normal control group, ^@^vs obese group, ^#^vs ZnONPs (5 mg/kg) at *p* < 0.05. ZnONPs: Zinc oxide nanoparticle.

According to results presented in Fig. 3, ZnONPs (5 mg/kg) administration to obese rats significantly (at *p* < 0.0001) reduced plasma MPC-1, resistin, ENA-78, TNF-α, IL6, and CRP levels by 23%, 53%, 49%, 48%, 39%, and 46% respectively. While ZnONPs (10 mg/kg) administration to obese rats mitigated (at *p* < 0.0001) these parameters by 37%, 65%, 72%, 63%, 50%, and 76%, respectively. There was a robust correlation between BMI and MCP-1.

### Cardiac and adipose tissue trace elements

**Figure 4:**
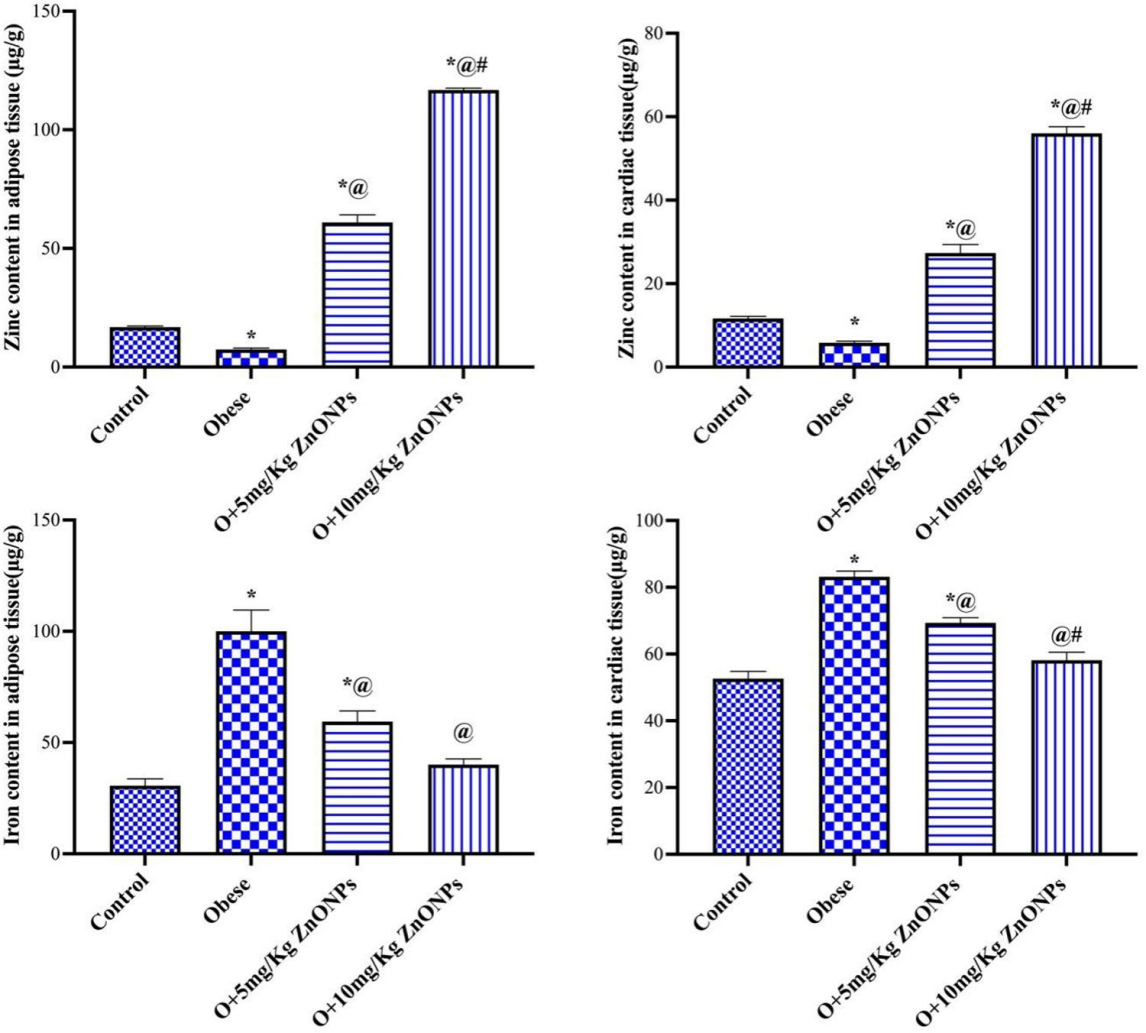
Effect of ZnONPs on cardiac and adipose tissue trace elements in obese rats. Each bar represents the mean ± SE of 8 rats. *vs normal control group, ^@^vs obese group, ^#^vs ZnONPs (5 mg/kg) at *p* < 0.05. ZnONPs: Zinc oxide nanoparticle.

The zinc content in cardiac and adipose tissue of obese rats decreased significantly (at *p* < 0.0001) by 50% and 55% compared to control values, respectively (Fig. 4). On the other hand, the zinc content in cardiac tissue of obese rats injected with ZnONPs (5 or 10 mg/kg) increased significantly (at *p* < 0.0001) by 4.7 or 9.6-fold, while the adipose zinc content elevated (at *p* < 0.0001) by 8.2 or 15.8-fold compared to the obese group, respectively. As shown in Fig. 4, our results indicated a significant increase in iron content of cardiac and adipose tissue of obese rats by 1.6 and 3 times of control value, respectively. However, the iron content decreased significantly (at *p* < 0.0001) by 17% or 30% in the cardiac tissue and by 41% or 60% in the adipose tissue for the ZnONPs (5 or 10 mg/kg) treated groups compared to the obese group. Moreover, the iron content in the two tissues of obese rats subjected to 10mg/kg ZnONPs did not change significantly relative to the control value.

### Plasma and cardiac tissue oxidative stress

**Figure 5:**
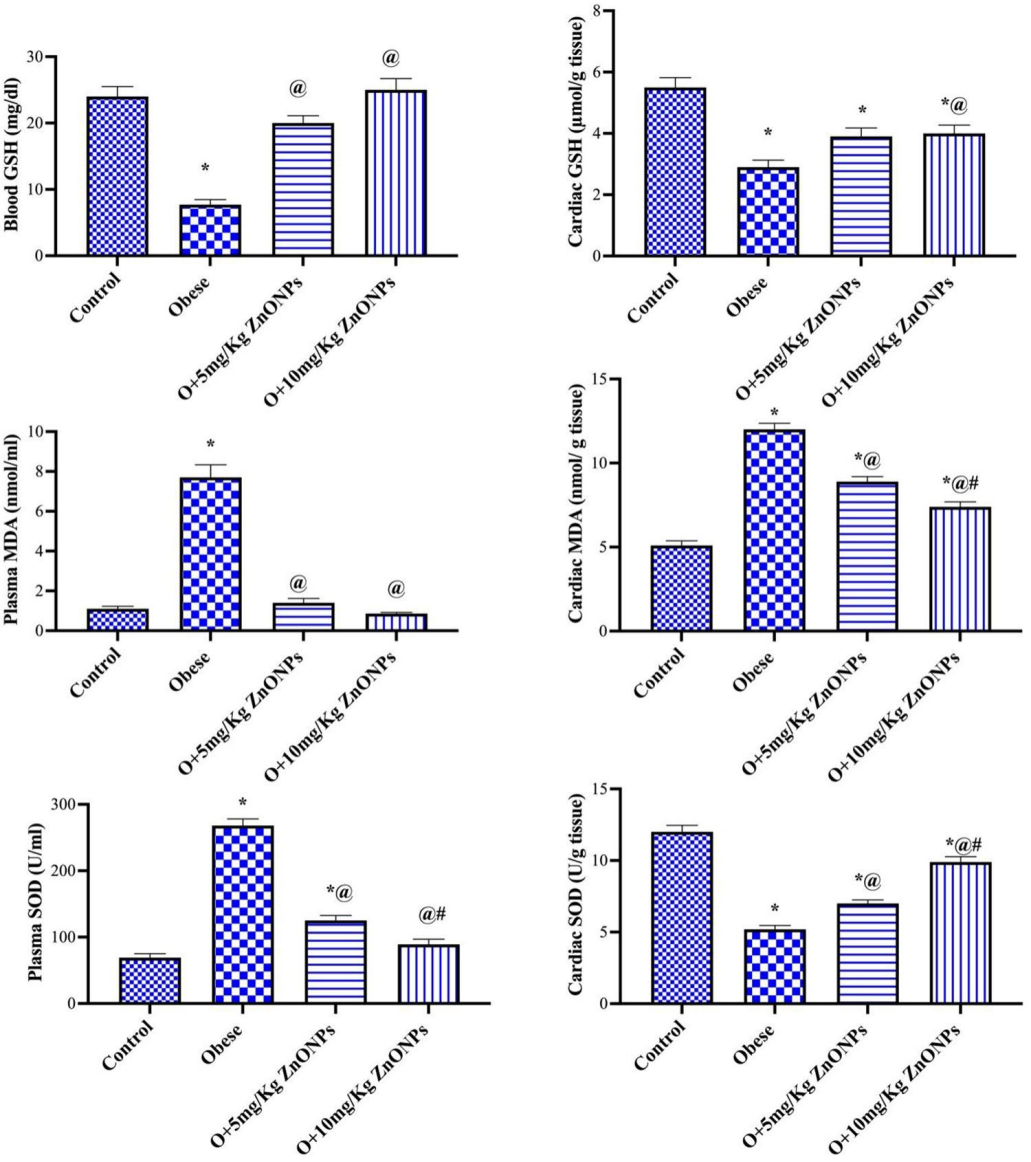
Effect of ZnONPs on plasma and cardiac tissue oxidative stress in obese rats. Each bar represents the mean ± SE of 8 rats. *vs normal control group, ^@^vs obese group, ^#^vs ZnONPs (5 mg/kg) at *p* < 0.05. ZnONPs: Zinc oxide nanoparticle.

**Figure 6:**
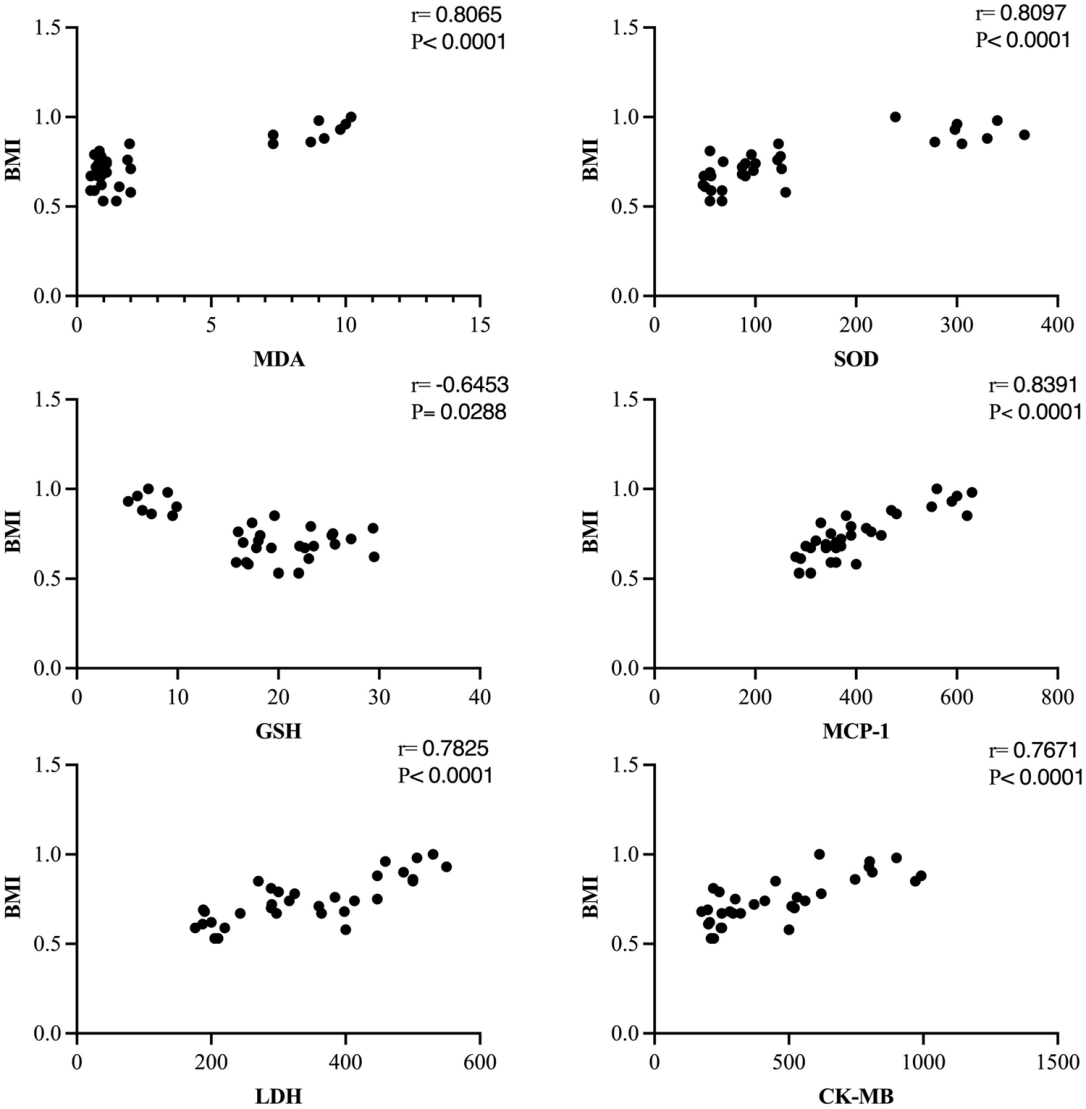
Correlation of BMI with plasma MDA, SOD, GSH, MCP-1, LDH, CK-MB.

Our results indicated a significant decrease in blood or cardiac tissue GSH level of the obese group (at *p* < 0.0001) by 68% or 47% and a significant increase (*p* < 0.0001) by 7 or 2.3-fold in MDA level, respectively (Fig. 5). The blood GSH and plasma MDA levels in ZnONPs (5 or 10 mg/kg) groups did not change significantly relative to the control value. The obese rats treated with ZnONPs (5 or 10 mg/kg) combated the decrease in blood GSH level (at *p* < 0.0001) by 2.6 or 3.2-fold, respectively, while only ZnONPs (10 mg/kg) hampered the increase of cardiac tissue MDA level (at *p* < 0.0489) 38% (Fig. 5) The activity of SOD exhibited a significant increase in plasma of the obese group (at *p* < 0.0001) by 3.8-fold. In contrast, the SOD activity decreased significantly in cardiac tissue (at *p* < 0.0001) by 56.6%. The treatment of obese rats with ZnONPs modulated the effect of high fat/sucrose diet on SOD (Fig. 5). It is worth mentioning the positive correlation between BMI and MDA and SOD. Moreover, there was a negative correlation between BMI and GSH (Fig. 6).

### Insulin resistance parameters

**Figure 7:**
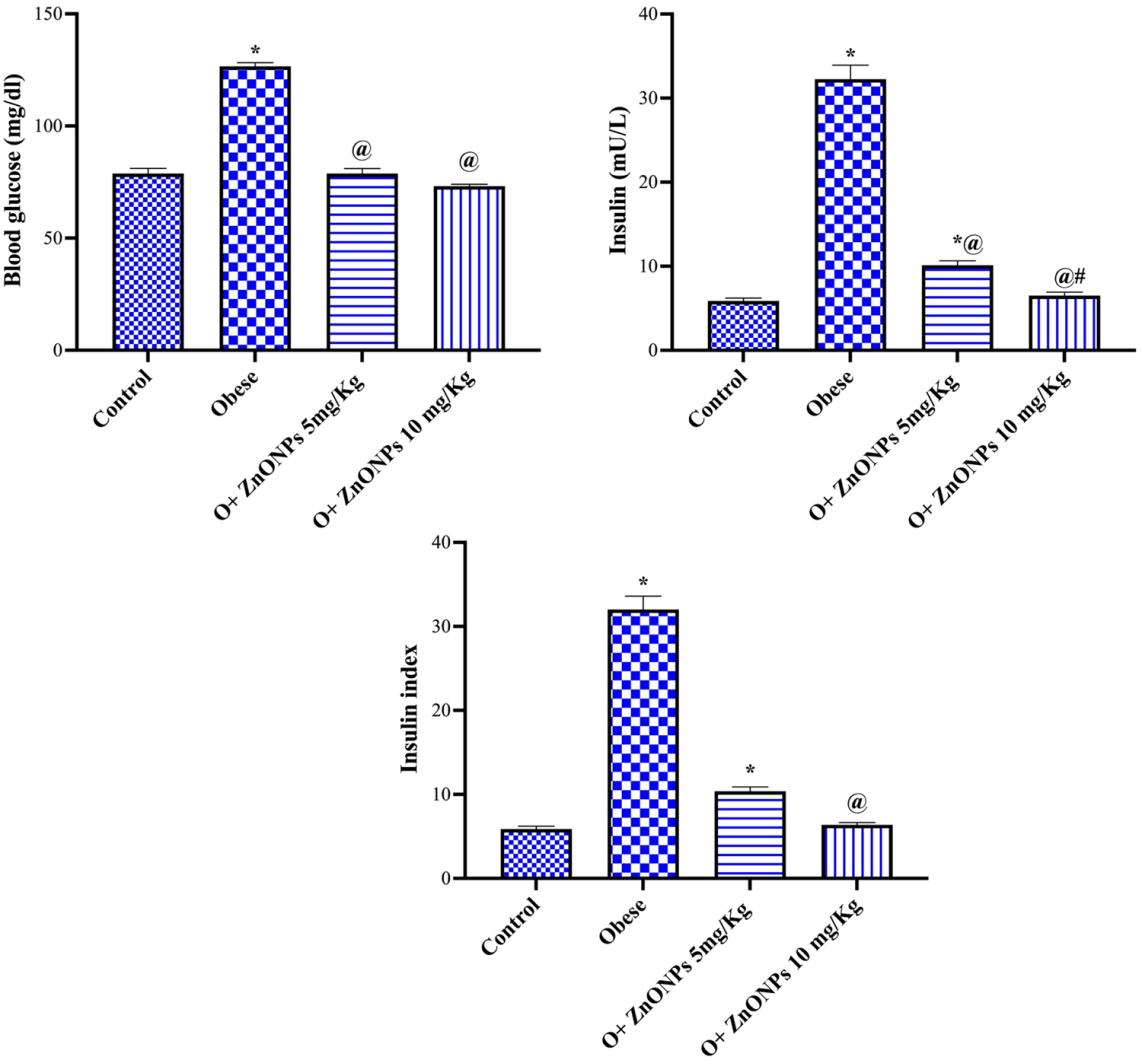
Effect of ZnONPs on insulin resistance parameters in obese rats. Each bar represents the mean ± SE of 8 rats. *vs normal control group, ^@^vs obese group, ^#^vs ZnONPs (5 mg/kg) at *p* < 0.05. ZnONPs: Zinc oxide nanoparticle.

The treatment of obese rats with ZnONPs at a dose level of 5 or 10 mg/Kg significantly mitigated the increase in plasma insulin (at *p* < 0.0001 or *p* < 0.0001) by 69% or 80% or glucose concentration (at *p* < 0.0001 or *p* < 0.0001) by 38% or 42% while only rats treated with ZnONPs 10 mg/Kg reduced insulin resistance index (at *p* < 0.0003) by 80% (Fig. 7), respectively. Mean values for insulin resistance index were 6.63 ± 0.39, 31.00 ± 1.49, 10.50 ± 0.77, and 6.75 ± 0.23 for control, obese, and ZnONPs (5 or 10 mg/kg), respectively.

### Cardiac enzymes biomarker

**Figure 8:**
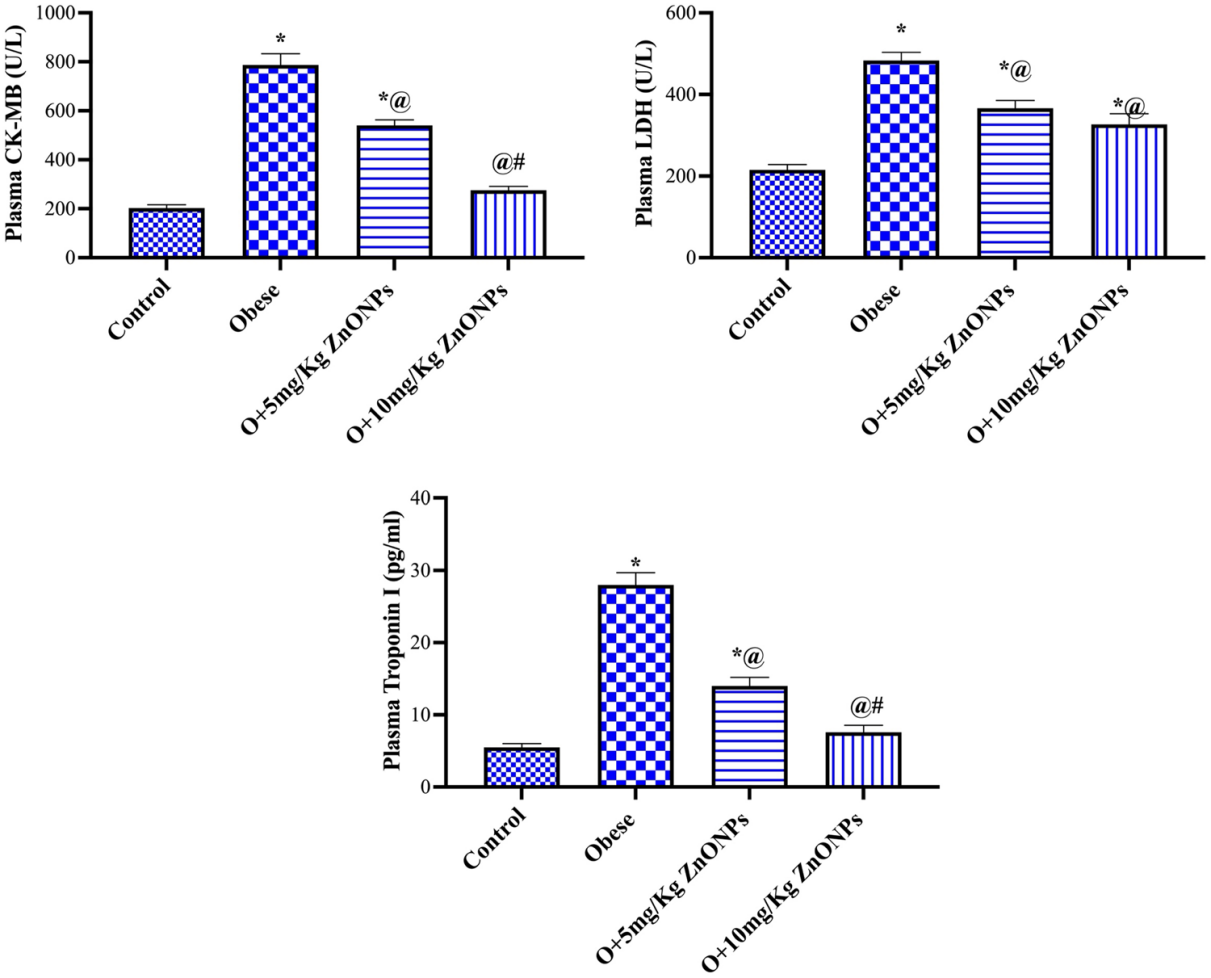
Effect of ZnONPs on cardiac enzymes biomarker in obese rats. Each bar represents the mean ± SE of 8 rats. *vs normal control group, ^@^vs obese group, ^#^vs ZnONPs (5 mg/kg) at *p* < 0.05. ZnONPs: Zinc oxide nanoparticle.

The obese rats manifested a significant (at *p* < 0.0001) rise in plasma LDH, CK-MB, and troponin levels by 124%, 3.9 and 5.1-fold (Fig. 8). The treatment of obese rats with ZnONPs (5 or 10 mg/kg) significantly (at *p* < 0.0001) lowered the level of LDH (at *p* = 0027 or *p* < 0.0001) by 24% or 33%, CK_MB (at *p* < 0.0001) by 31% or 65% and troponin (at *p* < 0.0001) by 50% or 73%, respectively. The levels of LDH were 215 ± 13, 483 ± 20,366 ± 19, and 326 ± 26 for control, obese, and ZnONPs (5 or 10 mg/kg), respectively, while mean values for Ck-MB were 203 ± 13, 787 ± 46, 540 ± 21.44, and 275 ± 16. The mean values for troponin were 5.5 ± 0.51, 28 ± 1.7, 14 ± 1.2, and 7.6 ± 0.96. There was a positive correlation between BMI and both LDH and CK-MB (Fig. 6).

### Histological study

**Figure 9:**
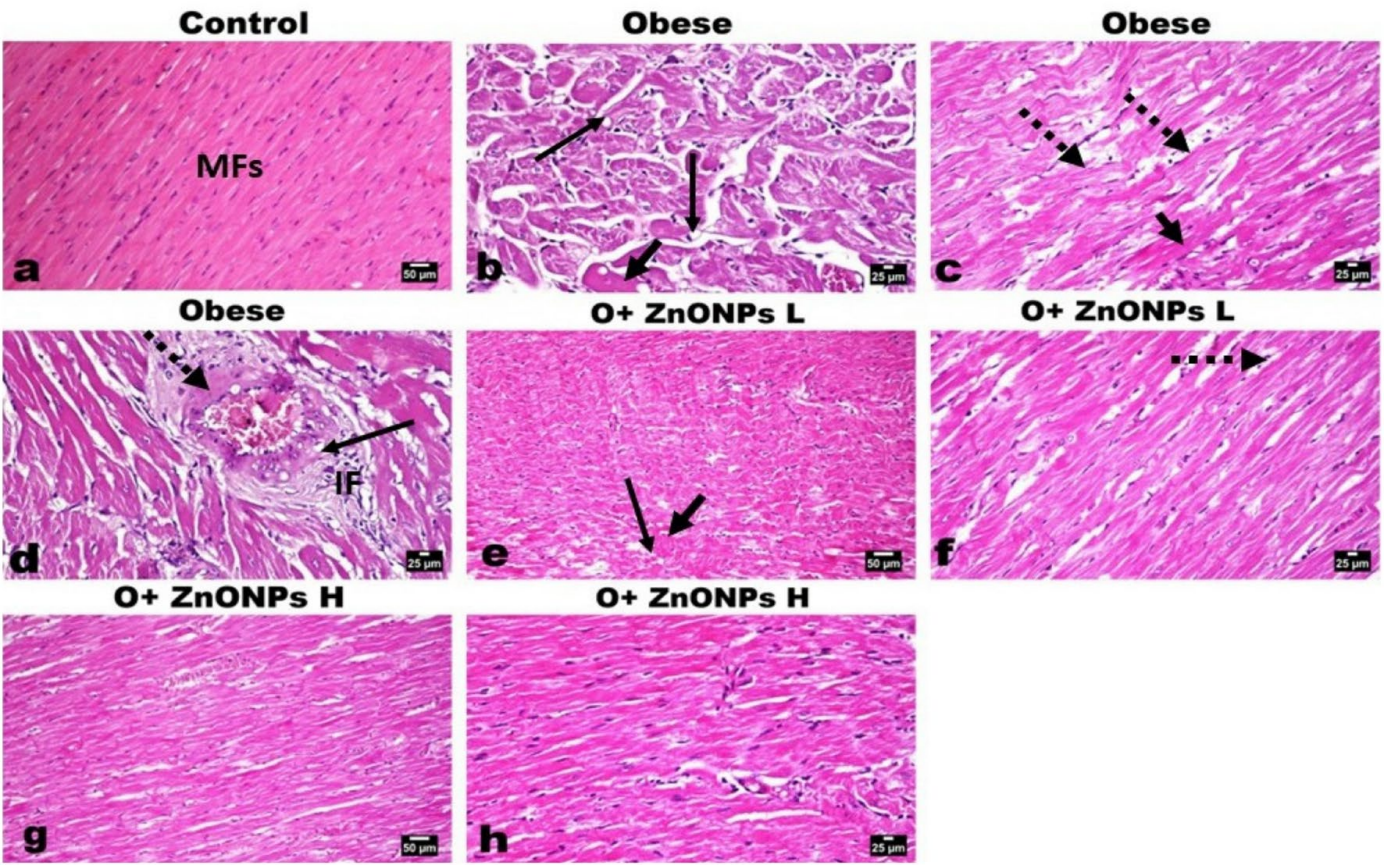
Photomicrographs of H&E-stained heart sections of various experimental groups. (**a**) Heart of control rat showing normal orientation ad striation of cardia muscles’ fibers (MFs). (**b–d)** Heart of obese rat showing marked degeneration (arrow), loss of striation, and scattered eosinophilia (short arrow) of the myofibers with increase intermuscular fat (dotted arrow). **(d)** The coronary vessels showing vacuolization of medial muscles (arrow) and focal fibroid necrosis (dotted arrow), and inflammatory cells infiltration (IF). (**e, f**) Heart of low dose ZnONPs administrated rat showing moderate degree of myofibers degeneration (arrow), mild eosinophilia (short arrow) with some intermuscular fat (dotted arrow). (**g, h)** Heart of high dose ZnONPs administrated rat showing good restoration of the cardiac muscle fibers with mild degeneration (arrow) and decreased intermuscular fat.

The cardiac muscles of control rats showed normal histological structure, orientation, and striation (Fig. 9a). While examination of the cardiac muscles of obese rats showed marked degenerative changes of the myofibers manifested by myofibers swelling, vacuolation, with scattered hyper-eosinophilia, loss of striation (Fig. 9b), fragmentation and sometimes rupture of the myofibers. The ruptured fibers are characterized by retraction caps, which are concavities at the free ends of the ruptured fibers. Infiltration of macrophages was seen in a focal manner phagocytosing the debris. Well as increased inter-muscular fat was also noticed (Fig. 9c). The blood vessels in the vicinity showed leukocytoclastic inflammation characterized by thickening and edema of their walls with focal fibroid necrosis and vacuolization of medial muscles (Fig. 9d). The administration of ZnONPs significantly decreased the pathological alterations observed in the cardiomyocytes in both the low (Fig. 9e, f) and high (Fig. 9g, h) dose groups, particularly with the later dose administration. Only mild degenerative changes and scattered necrotic muscle fibers with an apparent restoration of the vascular pathology.

**Figure 10:**
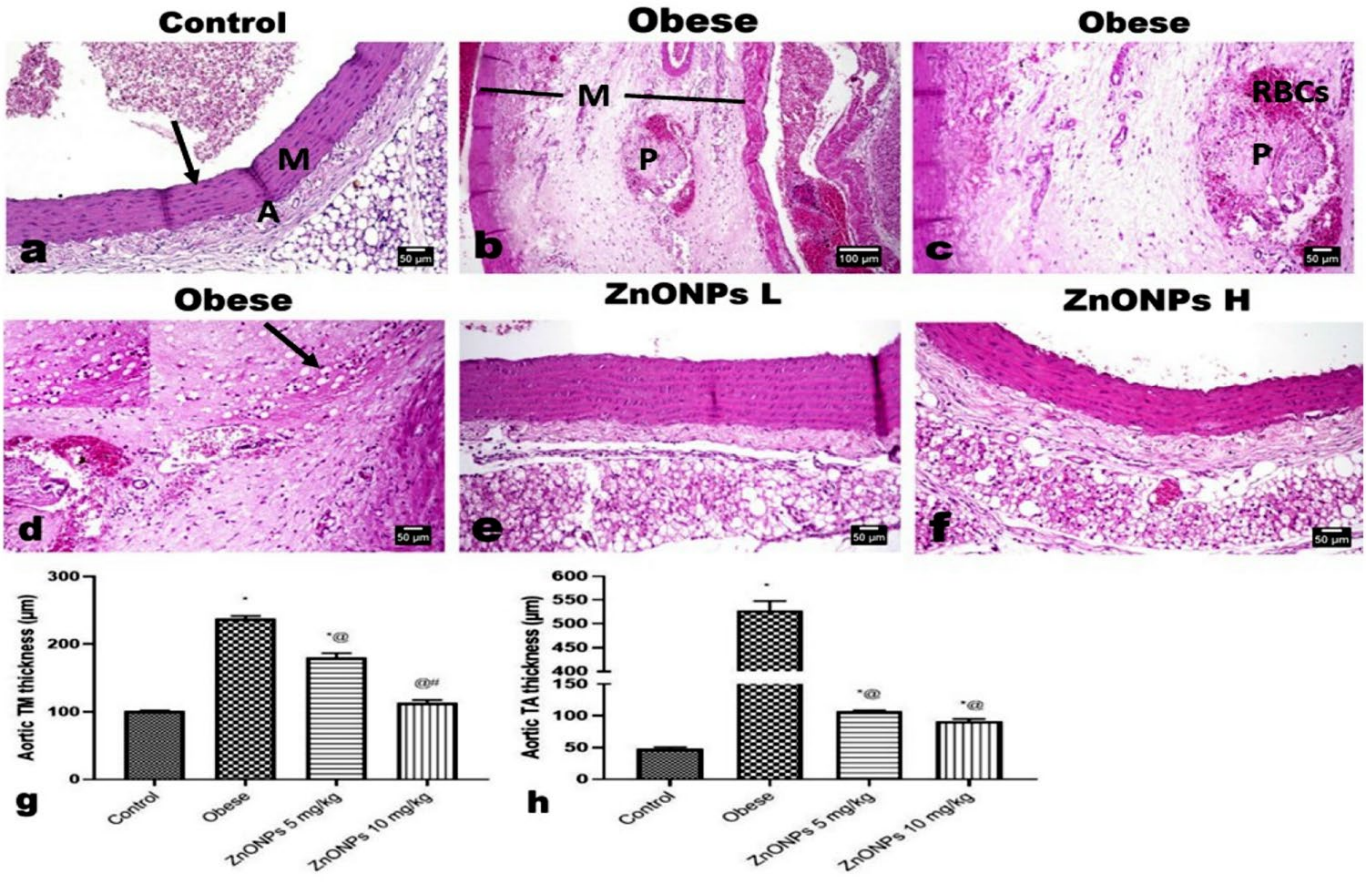
Photomicrographs of H&E-stained aorta sections. (**a)** Aorta of control rat showing normal histological structure of the aortic three layers; intima (arrow), media (M) and adventitia (A). (**b–d)** Aorta of obese rat showing splitting of the medial muscles (M) with congested vessels, free RBCs, mononuclear inflammatory cells, atherosclerotic plaque, and many lipophages (insert and arrow). Aortae of **(e)** low and **(f)** high doses ZnONPs administrated rats showing significant improvement in the histological changes with absence of any plaque as well as lipophages. (**g, h**) Marked reduction in the aortic tunica thickening. Each bar represents the mean ± SE of 8 rats. *vs normal control group, ^@^vs obese group, ^#^vs ZnONPs (5 mg/kg) at *p* < 0.05. ZnONPs: Zinc oxide nanoparticle.

**Figure 11:**
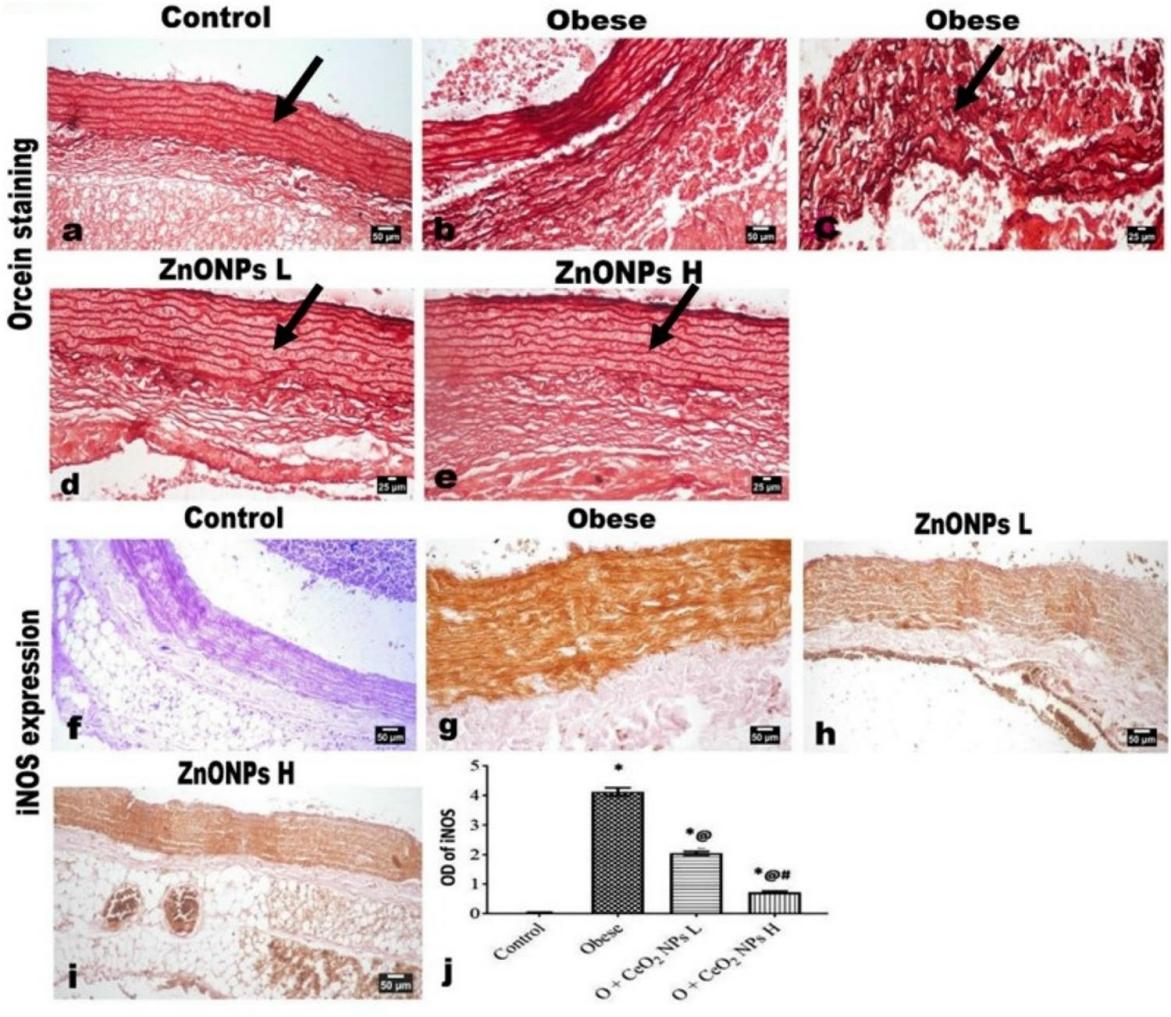
Photomicrographs of Orcein-stained aorta sections. showing (**a)** normal elastic lamellae (arrow). (**b, c**) Discontinues, disorganized, and fragmented elastic lamellae (arrow) in aorta of obese rat. **(d)** Low and **(e)** high doses ZnONPs administrated rats showing improvement in the deposition of elastic fibers. (**f–j)** Photomicrographs for the immunohistochemical expression of iNOS showing significant increased expression in obese rat’s aorta and significant decreased its expression in the aortic walls of obese model rats treated with ZnONPs at both low and high doses as quantified by image analysis of the optical density of the positive brown color. Each bar represents the mean ± SE of 8 rats. *vs normal control group, ^@^vs obese group, ^#^vs ZnONPs (5 mg/kg) at *p* < 0.05. ZnONPs: Zinc oxide nanoparticle.

Microscopic examination of the aorta of control rats showed the normal histological structure of endothelial cells of tunica intima (TI), tunica media (TM), and tunica adventitia (TA) (Fig. 10a) without any atherosclerotic lesion at all. At the same time, the aorta of rats of the obese group exhibited marked histological changes and thickening of the three tunicae with a marked increase of the periaortic fat as well as a reduction in the elastic fibers in the tunica of the aorta. The intima showed irregularity; the intimal endothelial cells showed swelling, cytoplasmic vacuolation, and focal desquamation with the attachment of mononuclear leukocytes to the denuded area. The TM showed splitting of the muscle fibers with congested vessels, free RBCs, mononuclear inflammatory cells, and an apparent atherosclerotic plaque (Fig. 10b) and inter-muscular edema inter-muscular edema (Fig. 10c). Many lipophages (foam cells) were evident (Fig. 10d) with extensive deposition of lipid at which the muscle fibers revealed marked vacuolation. The later alterations were conspicuously reduced with the administration of ZnONPs at low (Fig. 10e) and high (Fig. 10f) doses, particularly in the latter dose group, which showed a significant improvement in the histological changes. A profound reduction in the aortic tunica thickening was observed (Fig. 10g, h). Additionally, there was a significant improvement in the deposition of elastic fibers as evidenced by Orcein staining, which revealed multiple undulated continuous elastic lamellae in the aortae of control rats (Fig. 11a). While the aortae of obese rats showed somewhat straightening, discontinues, disorganized, and fragmented elastic lamellae (Fig. 11b, c). The ZnONPs administrated groups (Fig. 11d, e) revealed restoration of the elastic lamellae to an almost control-like structure in the high-dose group.

The examination of the periaortic adipose tissue revealed a conspicuous increase with a marked increase in the white adipocytes, which appeared more prominent than the other groups. Additionally, both brown and differentiating adipocytes were also increased in size. The administration of ZnONPs at two different doses showed pronounced amelioration expressed by decreased periaortic fat with decreased amount and size of white fat.

### An immunohistochemical expression both of iNOS in the aortae and leptin in the periaortic fat

The administration of ZnONPs resulted in significantly decreased expression of iNOS in the aortic walls of obese model rats, as presented in Fig. 11f–j, particularly in the high-dose administrated rats.

**Figure 12:**
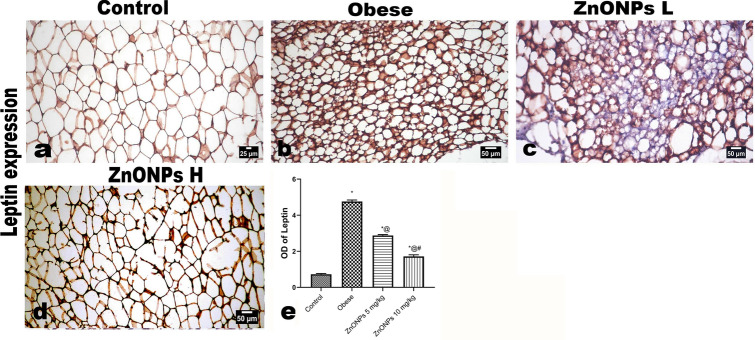
Photomicrographs of the immunohistochemical expression of leptin in periaortic fat showing: (**a**) positively stained thin rim of adipocytes’ cytoplasm. intense expression of leptin in various types of adipocytes, marked decreased leptin expression in ZnONPs treated groups. The positive brown color is quantified as optical density by image analysis software. Each bar represents the mean ± SE of 8 rats. *vs normal control group, ^@^vs obese group, ^#^vs ZnONPs (5 mg/kg) at *p* < 0.05. ZnONPs: Zinc oxide nanoparticle.

The periaortic fat from control rats showed a thin rim of the cytoplasm of the adipocytes, which were positively stained for leptin (Fig. 12a). While the periaortic fat of obese rats showed intense expression of leptin in various types of adipocytes (Fig. 12b). The expression of leptin markedly decreased in ZnONPs treated groups (Fig. 12c, d).

The scoring of leptin staining in both unilocular adipocytes and differentiating adipocytes was classified as follows; in control rats, it was classified as level I, while in obese, it was classified as level III. The treatment with ZnONPs significantly decreased leptin expression to levels ranging from levels II and I (Fig. 12e).

## Discussion

It is accepted that obesity is a risk factor for many diseases, including cardiovascular disease (CVD), insulin resistance, high blood pressure (HBP), and atherosclerosis^38^.

In the current study, The survey spectrum XPS spectra (Fig. 2a) showed the presence of C-1s, Zn-2p, and O-1s. The presence of C (C-1s) may be due to the adsorption on the sample's surface, as a residue, or during sample preparation. The C-1s XPS spectra (Fig. 2b) showed multiple peaks at 294.28, 292.15, 289.48, and 285.27 eV associated with C = C diffraction peak, C-O peak, O = C-O and C–C/C–H, respectively^37,38^.

The XP spectrum of O-1s (Fig. 2c) showed peaks at 531.06 eV and 532.84 eV associated with metal–oxygen bonds in the metal oxide and O–C, respectively^41^. The Zn-2p XPS spectra (Fig. 2d) of the nano compound showed Zn-2p_3/2_ and ZnO 2p_1/2_ characteristic peaks for ZnO at 1021.24 eV and 1044.19 eV, respectively, with a spin–orbit splitting value of ≈ 23 eV indicating the existence of Zn in the +2 oxidation state.^42^.

The sharp peaks in Fig. 2e indicate well-crystallized nanoparticles, which were matched by XRD reference codes 9011662 for the standard hexagonal wurtzite structure of ZnO with space group P 63 m c^43^. In this phase, two sub lattices of O^2−^ and Zn^2+^ are present where anions are present at the corners of a tetrahedron surrounded by four cations in a typical sp^3^ covalent bonding. To simplify the calculation of the unit cell parameters (a, b, and c) and the interplanar spacing d from hexagonal wurtzite structure, the planes (1 0 0), (0 0 2), and (1 0 1) were used. The values of unit cell parameters (a = b = 32.52 × 10^–2^ nm, c = 52.11 × 10^–2^ nm, and c/a = 1.60) indicate an ideal wurtzite crystal with a hexagonal close-packed lattice^44^. Crystallite sizes were determined using both HRTEM (Fig. 2f) and Debye–Scherrer Method. Both methods gave very close results (18.36 nm and 18.72 nm). In Debye–Scherrer Method, the average values of all peaks were used. The agglomerates of the nanoparticles can be seen in the TEM image (Fig. 2h). The zinc–oxygen bond length (1.98 Å) which slightly less than the known range (1.99 Å and 2.00 Å)^45^. EDX spectrum (Fig. 2g) only showed the presence of Zn and O, indicating the high purity of the nano zinc oxide.

Zinc plays a vital role in cardiovascular diseases by controlling lipid metabolism and inflammation^46^. Our findings indicated a significant decrease in weight gain, BMI, adipose tissue weight, and food intake in obese rats treated with ZnONPs. Furthermore, it was reported that zinc enhanced serotonin biosynthesis, which evoked satiety and lowered food intake^47^. Incongruous with our study, Khorsandi et al. (2019) noticed that daily administration of zinc to healthy obese adults results in a reduction of anthropometric measurements (body weight, BMI, waist circumferences)^48^, inflammatory markers, insulin resistance, and appetite. The present results signalized the influence of ZnONPs on adiponectin and leptin secretions, adipokines secreted by white adipose tissue. The improvement of anthropometric measurements or weight gain by ZnONPs may be attributed to the significant conservation of adiponectin and leptin concentrations. Obesity is characterized by a low level of adiponectin and a high level of leptin^49^. In addition, adiponectin decreases body weight gain via the enhancement of fatty acid oxidation by the muscle^50^.

The decrease in adipose tissue weight due to treating obese rats with ZnONPs led to a decline in the concentration of inflammatory markers secreted from adipose tissue. The elevation in body weight in obesity, which results in exaggerated fat accumulation, has been a critical factor in the pathogenesis of several diseases, including cardiovascular disease^51^. In the present work, ZnONPs exert anti-inflammatory properties in obese rats, evidenced by decreased TNF-α, CPR, IL6, resistin, and MCP-1. Moreover, ZnONPs enhanced adiponectin levels, improved cardiovascular functions, and displayed anti-inflammatory effects^52^. Oxidative stress and inflammation markers were increased, and antioxidant levels were decreased in obese subjects^53^. Our results indicated a significant increase in glucose, cholesterol, triglycerides, leptin, blood pressure, and inflammatory markers that may result in oxidative stress. The possible sources of oxidative stress in obesity include hyperglycemia^54^, Hypercholesterolemia^55^, chronic inflammation^56^, hyperleptinemia^9^, and hypertension^57^.

Other possible contributors to oxidative stress in obesity were elevated cardiac and adipose iron content and decreased zinc concentration observed in the present work. The link between iron overload and oxidative stress associated with metabolic dysfunction, type 2 diabetes, and CVD was reported^58,59^. The increase of iron content in adipose or heart tissues in obese rats of the present study may be attributed to the rise of leptin level, which elevates hepcidin level that induces cellular retention of iron through reducing iron export from different tissues^60^.

Here, the obese rats exhibited cardiac oxidative stress as affirmed by the elevation of cardiac MDA level and a decrease of both GSH and SOD levels suggesting oxidative stress involved in obesity-induced myocardial injury. GSH and SOD are powerful endogenous antioxidants that protect against reactive oxygen species, reducing oxidative stress^61,62^. Malondialdehyde is a lipid peroxidation product linked with oxidative stress-associated cardiac dysfunction^63^.

Here, the cardiac muscles of obese rats showed myofibers swelling and vacuolation, with scattered hyper-eosinophilia, loss of striation, fragmentation, and sometimes rupture of the myofibers. The pathological changes in the heart muscle of obese rats can be attributed to increased cardiac oxidative stress^64^.

Moreover, insulin resistance and hypertension observed in the present obese rats may participate in cardiovascular disease. IR can provoke hyperglycemia, which excites oxidative stress and inflammatory response, leading to cell damage. Also, IR plays a vital role in the progress of dyslipidemia, a primary metabolic disorder affecting endothelial dysfunction^3^. Our results indicated a significant increase in systolic and diastolic blood pressure in obese rats. Hypertension is critical in the progress of left ventricle hypertrophy leading to diastolic and systolic dysfunctions followed by heart failure^65^. Both high BP and high total cholesterol can synergistically increase the risk of coronary heart disease^66^.

Zinc is essential for the conservation of the structure and function of the cells. Any disturbance in zinc homeostasis can lead to diseases like cardiovascular disease. There is a significant link between low serum zinc levels and heart failure^67^. Our findings indicated that treating obese rats with ZnONPs decreased the pathological alterations observed in the cardiomyocytes. The decrease in the pathological changes can be referred to as the direct effect of ZnONPs as antioxidants deduced from a lowering cardiac MDA and iron contents or enhancement of cardiac GSH and SOD. The antioxidant properties of ZnONPs were reported^22^.

Moreover, ZnONPs have indirect mechanisms to alleviate cardiac pathology in obesity by reducing blood pressure, insulin resistance, dyslipidemia, and leptin level or restoring adiponectin concentration. Leptin is a primary contributor to numerous cardiovascular risks connected to obesity. It promotes hypertension, vascular remodeling, ROS generation, and atherosclerosis. Otherwise, adiponectin is a cardioprotective hormone that reduces left ventricular and vascular hypertrophy^68^. In addition, it was reported that zinc plays an intrinsic role in relieving the development of metabolic syndrome, regulating cytokine expression, inhibiting inflammation, and reducing oxidative stress^69^. Our results showed that ZnONPs have anti-inflammatory effects, as evidenced by a decrease in levels of CRP, IL6, and TNF-α in obese rats, which may participate in decreasing cardiac pathology. The increase in the level of both IL6 and TNF-α was reported to enhance iron overload in tissues due to elevated hepcidin expression^70,71^. A positive association between oxidative stress, iron overload, and insulin resistance was reported^72^.

The significant role of ZnONPs in alleviating cardiovascular disease was affirmed by combating the increase in troponin and creatine phosphokinase observed in obese rats routinely used as a diagnostic tool for myocardial injury and coronary syndrome^73^. It appears that the treatment of obese rats with ZnONPs inhibits the progression of endothelial dysfunction, as evidenced by a reduction in the aortic tunica thickening, fibroid necrosis in the aorta, foam cells, and plasma levels of MCP-1, resistin, and CRP. MCP-1, an efficient chemoattractant for monocytes, drives mononuclear phagocytes that accumulate in the newly formed atheroma. Hence, it plays an essential role in early atherogenesis^74^. It was found that CRP harms the endothelial cells directly, and it correlates with atherosclerosis^75^. Moreover, ZnONPs resist the decrease in plasma nitric oxide observed in obese rats. Nitric oxide has vital roles in cardio-protection by controlling blood pressure and vascular tone, suppressing platelet aggregation and leukocyte adhesion, and inhibiting smooth muscle cell proliferation^76^. Zinc supplementation can prevent endothelial dysfunction via several mechanisms, such as the inhibition of the increase in NF-κB-induced inflammatory markers, the induction of an increase in eNOS expression levels and NO availability, the activation of PPAR receptor, and the inhibition of TNFα activation-induced apoptosis^77^. It appears that the virtue of ZnONPs in lowering body and adipose tissue weight alleviates risk factors linked to cardiovascular disease-associated obesity. It was reported that significant body weight loss in obese individuals decreases chronic inflammation and serum hepcidin levels, resulting in improved iron status due to increased iron absorption^78^.

It appears that ZnONPs combat the development of cardiac pathology associated with obesity directly through their antioxidant properties or indirectly via lowering oxidative stress, inflammatory markers, and iron overload or enhancement of cardiac SOD and GSH. Moreover, ZnONPs reduced insulin resistance, BMI, and body weight gain in obese rats, which may decrease cardiac pathology. Furthermore, ZnONPs restrained the increase of MCP-1, lipids, and blood pressure in obese rats, which is considered the initiation of atherosclerosis. Additionally, ZnONPs reduced aortic tunica thickening and the amount and size of periaortic fat, which may indicate their role in preserving the aorta in normal function and preventing pre-atherosclerosis. These results suggest that ZnONPs may be a novel therapeutic agent for cardiovascular disease.

## Data Availability

The datasets generated during and/or analyzed during the current study are available from the corresponding author on reasonable request.
