# Author Correction: Zinc nanoparticles ameliorated obesity-induced cardiovascular disease: role of metabolic syndrome and iron overload

**DOI:** 10.1038/s41598-024-53947-8

**Published:** 2024-02-14

**Authors:** Samir A. E. Bashandy, Ahmed M. A. El-Seidy, Fatma A. A. Ibrahim, Sahar S. Abdelrahman, Sherif A. Abdelmottaleb Moussa, Marawan A. ElBaset

**Affiliations:** 1 Pharmacology Department, National Research Centre, 33 El-Bohouth St., Dokki, P.O. 12622, Cairo, Egypt; 2 Inorganic Chemistry Department, National Research Centre, 33 El-Bohouth St., Dokki, P.O. 12622, Cairo, Egypt; 3 Biophysics Group, Department of Biochemistry, National Research Centre, 33 El-Bohouth St., Dokki, P.O. 12622, Cairo, Egypt; 4 Pathology Department, Faculty of Veterinary Medicine, Cairo University, Cairo, Egypt

Correction to: *Scientific Reports* 10.1038/s41598-023-42550-y, published online 25 September 2023

The original version of this Article contained an error in Figure 2, panel d, where the value “ZnO 2p_1/2_” was incorrectly given as “ZnO 2p_5/2_”. The original Figure 2 and accompanying legend appear below.

**Figure 2:**
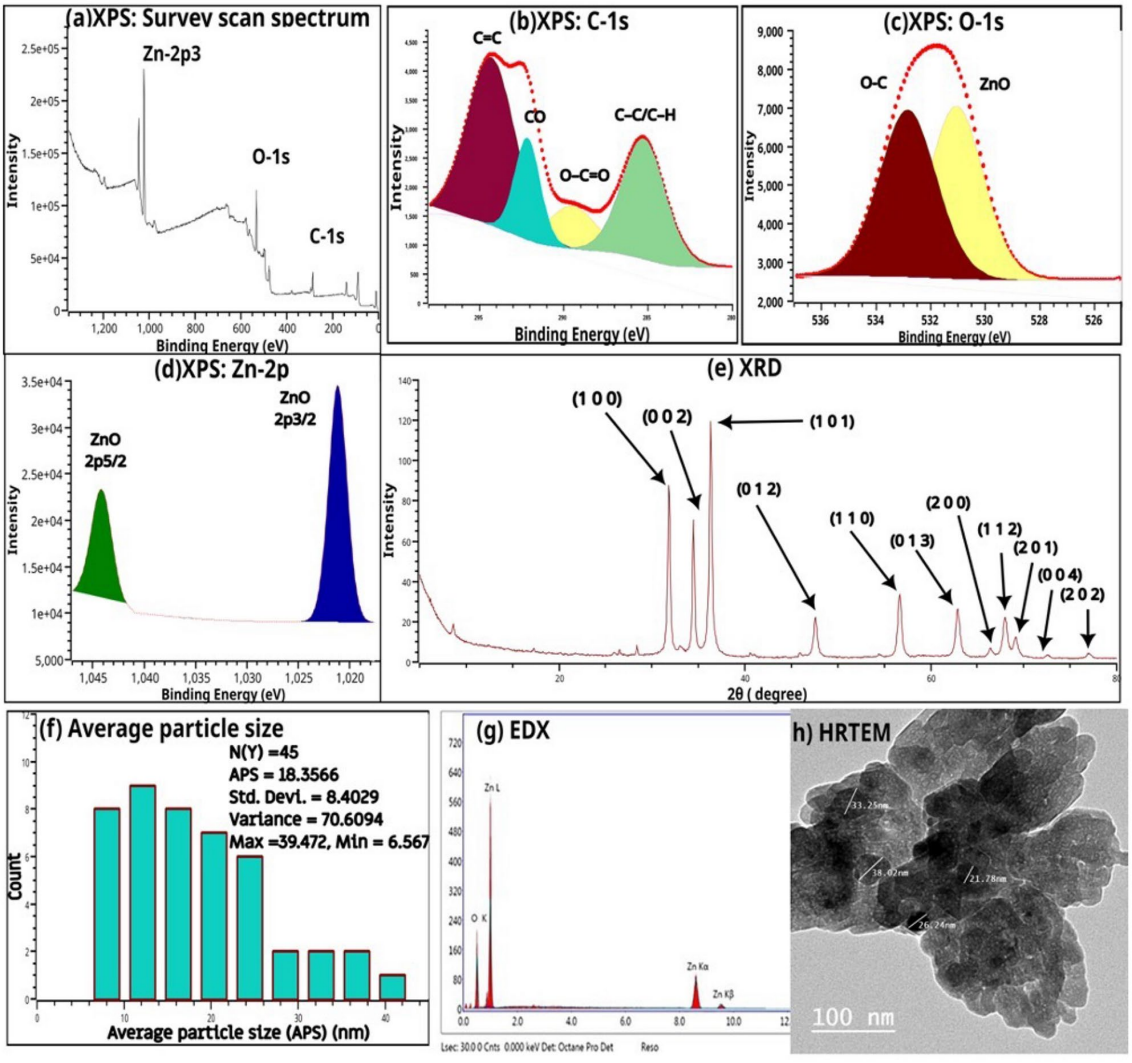
(**a**) XPS Survey scan spectra of nano zinc oxide, (**b**) C-1s XPS spectra, (**c**) O-1s XPS spectra, (**d**) Zn-2p XPS spectra, (**e**) XRD patterns of nano zinc oxide, (**f**) Particle diameters distributions, (**g**) EdX and (**h**) HRTEM of nano zinc oxide.

In addition, Figure 12d contained an error. Due to a mistake during figure assembly an image from the wrong condition was used. The original Figure 12 and accompanying legend appear below.

**Figure 12:**
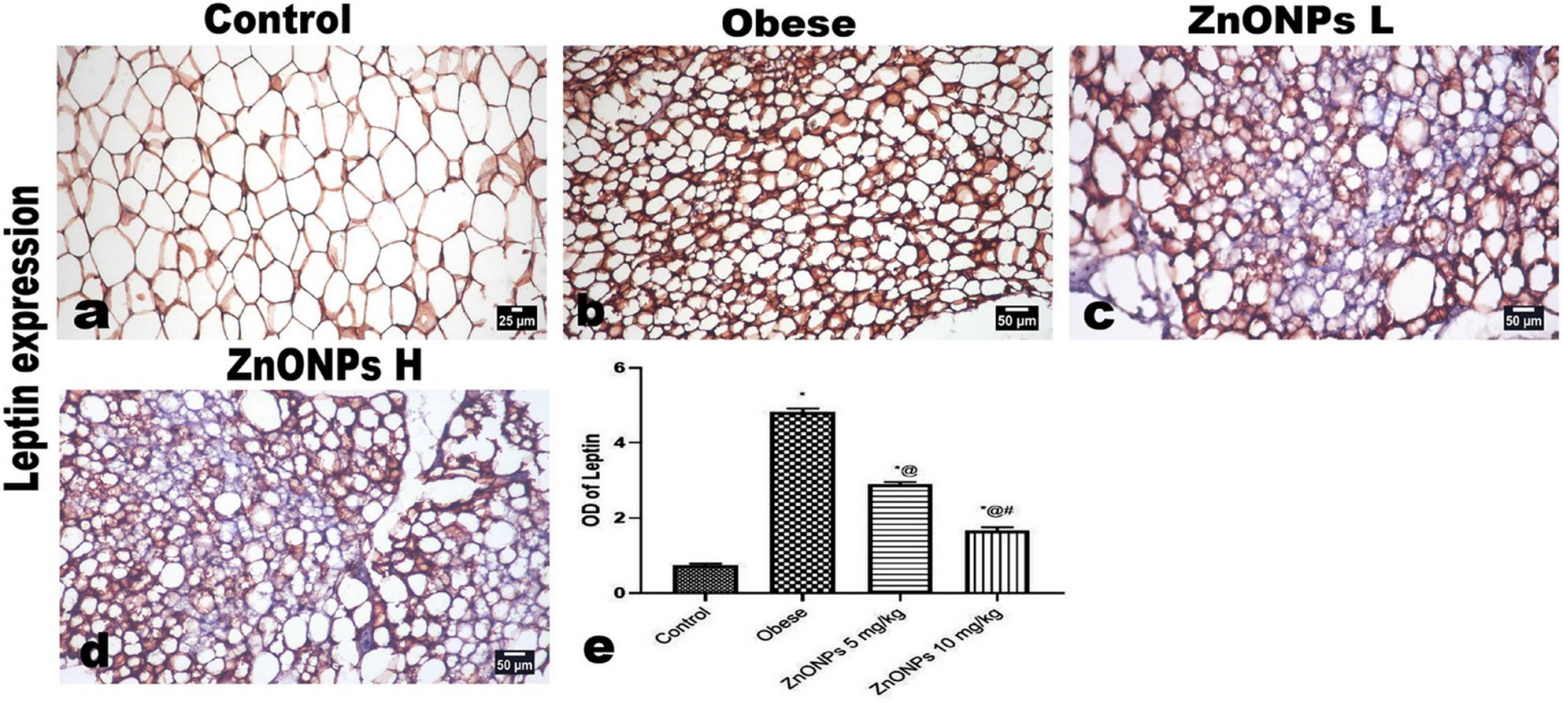
Photomicrographs of the immunohistochemical expression of leptin in periaortic fat showing: (**a**) positively stained thin rim of adipocytes’ cytoplasm. intense expression of leptin in various types of adipocytes, marked decreased leptin expression in ZnONPs treated groups. The positive brown color is quantified as optical density by image analysis software. Each bar represents the mean ± SE of 8 rats. *vs normal control group, ^@^vs obese group, ^#^vs ZnONPs (5 mg/kg) at *p* < 0.05. ZnONPs: Zinc oxide nanoparticle.

Additionally, Table 2 contained errors. Due to a mistake in preparing the table, the data for Cholesterol, Triglycerides, HDL, and LDL were accidentally copied from^1^. The original Table 2 appears below as Table 1.

**Table 1:** Plasma lipid profile (mg/dl), adipocyte hormones (µg/L), nitric oxide (µmol/ml) levels and blood pressure (mmHg) of obese rats treated with zinc oxide nanoparticles.

Parameter	Treatment			
	Control	Obese	O + ZnONPs 5 mg/kg	O + ZnONPs 10 mg/kg
Cholesterol	90.63 ± 2.76	197.60 ± 4.97*	131.50 ± 2.77*^@^	87.13 ± 6.58^@#^
Triglycerides	66.88 ± 1.66	231.50 ± 13.63*	200.50 ± 14.47*	168.60 ± 12.86*^@^
HDL	37.57 ± 2.28	12.00 ± 0.54*	28.13 ± 2.35*^@^	40.00 ± 2.41^@#^
LDL	21.88 ± 1.27	87.38 ± 1.90*	43.63 ± 2.21*^@^	25.25 ± 0.88^@#^
Atherogenic index	0.26 ± 0.02	1.31 ± 0.03*	0.87 ± 0.02*^@^	0.66 ± 0.04*^@#^
Leptin	1.87 ± 0.15	3.11 ± 0.15*	2.19 ± 0.25*^@^	1.94 ± 0.19^@#^
Adiponectin	196 ± 10.31	51.13 ± 1.49*	151.88 ± 8.33*^@^	188.13 ± 8.80^@#^
Nitric oxide	40.80 ± 1.16	17.88 ± 0.78*	26.00 ± 1.05*	38.50 ± 1.68^@#^
Systolic BP	117.87 ± 2.13	145.76 ± 2.95*	130.38 ± 1.06*^@^	121.62 ± 1.89 ^@^
Diastolic BP	81.13 ± 1.62	102.50 ± 2.60*	88.38 ± 1.44^@^	80.75 ± 2.23^@^

Each value represents the mean of 8 animals ± SE. Statistical analysis was performed using one-way ANOVA followed by Tukey–Kramer multiple comparisons test (*vs control group, ^@^vs obese group and ^#^vs ZnONPs 5 mg/kg) at *p* < 0.05. O: Obese.

[1] El-Seidy, A. M. A., Bashandy, S., Ibrahim, F., Abd El-Rahman, S., Farid, O., Moussa, S., El-Baset, M. Zinc oxide nanoparticles characterization and therapeutic evaluation on high fat/sucrose diet induced-obesity. *Egyptian Journal of Chemistry*, 2022; 65(9): 497–511. doi: 10.21608/ejchem.2022.112166.5113

Furthermore, in the Discussion section,

“The Zn-2p XPS spectra (Fig. 2d) of the nano compound showed Zn-2p_3/2_ and Zn-2p_5/2_ characteristic peaks for ZnO at 1021.24 eV and 1044.19 eV, respectively, with a spin–orbit splitting value of ≈ 23 eV indicating the existence of Zn in the +2 oxidation state^42^.”

now reads:

“The Zn-2p XPS spectra (Fig. 2d) of the nano compound showed Zn-2p_3/2_ and ZnO 2p_1/2_ characteristic peaks for ZnO at 1021.24 eV and 1044.19 eV, respectively, with a spin–orbit splitting value of ≈ 23 eV indicating the existence of Zn in the +2 oxidation state^42^.”

The original Article has been corrected.
